# Anticancer activity of triterpene glycosides from the sea star *Solaster pacificus*

**DOI:** 10.1038/s41598-025-12914-7

**Published:** 2025-08-04

**Authors:** Sergey A. Dyshlovoy, Jessica Hauschild, Malte Kriegs, Konstantin Hoffer, Olga Y. Burenina, Nadja Strewinsky, Timofey V. Malyarenko, Alla A. Kicha, Natalia V. Ivanchina, Valentin A. Stonik, Markus Graefen, Carsten Bokemeyer, Gunhild von Amsberg

**Affiliations:** 1 Laboratory of Experimental Oncology, Department of Oncology, Hematology and Bone Marrow Transplantation With Section Pneumology, Hubertus Wald-Tumorzentrum, University Medical Center Hamburg-Eppendorf, Martinistrasse 52, 20251 Hamburg, Germany; 2 Department of Radiotherapy and Radiation Oncology, Hubertus Wald Tumorzentrum – University Cancer Center Hamburg (UCCH), University Medical Center Hamburg-Eppendorf, Martinistrasse 52, 20251 Hamburg, Germany; 3 UCCH Kinomics Core Facility, Hubertus Wald Tumorzentrum – University Cancer Center Hamburg (UCCH), University Medical Center Hamburg-Eppendorf, Martinistrasse 52, 20251 Hamburg, Germany; 4 Center of Molecular and Cellular Biology, Bolshoy Blvd 30 bld1, Moscow, Russian Federation 121205; 5 G.B. Elyakov Pacific Institute of Bioorganic Chemistry, Far Eastern Branch of the Russian Academy of Sciences, Prospekt 100-let Vladivostoku 159, Vladivostok, Russian Federation 690022; 6 School of Advanced Engineering Studies, Institute of Biotechnology, Bioengineering and Food Systems, Far Eastern Federal University, Russky Island, Ajax Bay, 10, Vladivostok, Russian Federation 690922; 7 Martini-Klinik, Prostate Cancer Center, University Hospital Hamburg-Eppendorf, Martinistrasse 52, 20251 Hamburg, Germany

**Keywords:** Triterpene glycosides, Prostate cancer, *Solaster pacificus*, Marine natural compounds, P-glycoprotein, MAPK, Drug resistance, Prostate cancer, Biochemistry, Cancer, Drug discovery

## Abstract

Marine triterpene glycosides are known to exhibit significant anticancer activity. We investigated pacificusoside C and cucumariosides C_1_ and C_2_ isolated from a sea star *Solaster pacificus* in prostate cancer models with varying drug resistance and in non-cancerous cells in vitro. Cucumarioside C_1_ showed selectivity comparable to cisplatin, whereas the other compounds were less selective. Cucumarioside C_1_ induced apoptosis and enhanced cytotoxic effects of cisplatin, carboplatin, docetaxel, and cabazitaxel, making it a potential candidate for combination therapy. All three glycosides were active in docetaxel-resistant cells and were neither inhibitors nor substrates of P-glycoprotein, indicating P-glycoprotein-independent activity. To explore the mechanism of anticancer activity of cucumarioside C_1_, we performed functional kinome profiling of treated 22Rv1 cells, predicting activation of kinases involved in stress response and survival (IKKα, IKKβ, IKKε), necroptosis (MLKL), metabolism (GCN2, PDK1), cytoskeletal dynamics (RHOK), mitophagy (PINK1), apoptosis and cell cycle regulation (PITSLRE), and immune modulation (COT). Notably, only MAP kinases p38 and ERK1/2 were predicted to be specifically inhibited, that was further validated by Western blotting. These findings may potentially explain previously reported anticancer effects of cucumarioside C_1_ and related marine glycosides. To our knowledge, this is the first study to report triterpene glycosides’ effects on the kinome of cancer cells.

**Supplementary Information:**

The online version contains supplementary material available at 10.1038/s41598-025-12914-7.

## Introduction

Prostate cancer is the most frequently diagnosed malignancy in men, with an annual incidence exceeding 1.3 million cases worldwide. It ranks among the top five causes of cancer-related mortality globally^1^. While advanced prostate cancer in the hormone-sensitive setting can often be controlled with androgen deprivation therapy (ADT) in combination with androgen receptor (AR) signaling pathway inhibitors (ARPI), with or without the addition of chemotherapy for several years, disease progression to castration resistance eventually occurs in the majority of patients^2^. Here, live prolonging therapies including ARPIs (enzalutamide or abiraterone), PARP inhibitors (olaparib), taxane-based chemotherapy (docetaxel or cabazitaxel), and radioligandtherapy can be offered^3,4^. With an increasing number of therapy lines, resistance develops more rapidly, often leading to a shorter treatment duration. The primary mechanisms of therapy resistance include AR alterations (AR splice variants, mutations, amplifications), a shift to AR-independent growth, overexpression of efflux transporters such as P-glycoprotein, alterations in tubulin dynamics, epithelial-to-mesenchymal transition (EMT), modifications to the tumor microenvironment, as well as activation of pro-survival pathways, including PI3K/AKT/mTOR, JAK/STAT, and TGF-β^5^. Some patients develop a particularly aggressive form called aggressive variant of prostate cancer (AVPC). AVPCs are highly metastatic and resistant to most conventional therapies^6^. Therapeutic options for AVPC include platinum-based agents (e.g. cisplatin and carboplatin); however, due to the therapy’s limited efficacy, the disease ultimately progresses, leading to the patient’s demise. Therefore, despite recent progress in prostate cancer therapy, there is an urgent need for novel therapeutic strategies for managing drug-resistant prostate cancer.

Marine-derived bioactive compounds represent a promising reservoir for novel anticancer agents^7,8^. Many naturally occurring substances, even those with well-characterized structures, continue to reveal novel biological activities and targets in both in vitro and in vivo models^9^. Marine triterpene glycosides are a class of secondary metabolites found mainly in sea cucumbers (holothurians) and marine sponges^10^. These molecules are characterized by a broad spectrum of biological activities, including cytotoxic, ichthyotoxic, antimicrobial, antiviral, radioprotective, and immunomodulatory properties. Triterpene glycosides are currently used in various pharmaceutical formulations (e.g. vaccine adjuvant QS-21), functional food products, dietary supplements, and cosmetic applications^11,12^. Triterpene glycosides are occasionally isolated from sea stars^13,14^. These secondary metabolites are rarely produced by sea stars themselves and are likely acquired through their diet. The sea star *Solaster pacificus* was identified as a rich source of various triterpene glycosides, as this echinoderm species primarily feeds on holothurians^15–17^. It is also worth noting that another group of oligoglycosides, steroidal saponins, commonly called asterosaponins, was also found in sea stars^18^.

The antitumor potential of marine-derived triterpene glycosides is of particular interest. The anticancer activity of these natural compounds is primarily mediated via two major mechanisms. First, their membranotropic properties enable the disruption of cancer cell membranes, which further triggers apoptosis executed via both intrinsic and extrinsic pathways^19,20^. Second, these compounds can modulate several oncogenic signaling pathways, including NF-κB, PI3K/Akt/mTOR, MAPK, and VEGF/VEGFR, thereby inducing cell cycle arrest, inhibition of proliferation, and suppression of angiogenesis and metastasis^20–22^. Structure–activity relationship (SAR) studies have revealed that both the glycone (sugar moieties) and aglycone side chains are critical determinants of biological activity. In particular, the pattern and degree of sulfation significantly influence cytotoxic potency, selectivity, and the ability to interact with cellular membranes^10,23^.

Previous studies have demonstrated that these compounds inhibit the proliferation of various cancer types, with some showing activity in prostate cancer models. Thus, triterpene saponins cercodemasoides A–E (sea cucumber *Cercodemas anceps*), stichorrenosides A–D (sea cucumber *Stichopus horrens*), cucumarioside A_2_-2 (sea cucumber *Cucumaria japonica*), and echinoside A (sea cucumber *Actinopyga echinites*) exhibited cytotoxic activity in prostate cancer models both in vitro and in vivo^24–26^. Additionally, frondoside A (sea cucumber *Cucumaria frondosa*) was found to suppress prostate cancer tumor growth, to inhibit metastatic spread to the lungs, as well as to decrease the number of circulating tumor cells in the blood via modulation of cell cycle, apoptosis, and autophagy, as well as by stimulation of anticancer immunity^27^. Saponins are known to exert their cytotoxic effects primarily by targeting the cholesterol of the cellular membrane, forming complexes, and therefore physically disrupting the membrane permeability and integrity^28^. However, evidence suggests that saponins target additional biological pathways relevant to their anticancer activity^29^.Triterpene glycosides from the sea star *Solaster pacificus* have demonstrated anticancer and cancer-preventive properties. For example, pacificusoside M effectively suppressed the colony formation of colorectal carcinoma HCT 116 and breast cancer MDA-MB-231 cells at a non-toxic concentrations. Additionally, pacificusoside M showed a significant anti-invasive potential in an in vitro model^17^. Pacificusoside D and cucumarioside D (sea star *S. pacificus*) exhibited potent cytotoxicity against human melanoma SK-MEL-2, SK-MEL-28, and RPMI-7951 cell lines, and could inhibit a colony formation of cancer cells at non-toxic concentrations. Both compounds at their non-toxic concentrations inhibited neoplastic cell transformation of JB6 Cl41 cells induced by epidermal growth factor (EGF), 12-*O*-tetradecanoilphorbol-13-acetate (TPA), and ionizing radiation (X-rays and UVB)^16^. Finally, pacificusoside C, cucumariosides C_1_ and C_2_ from the sea star *S. pacificus* strongly suppressed the colony formation of the HT-29, RPMI-7951, and MDA-MB-231 cells at nontoxic concentrations^15^. However, the mechanism of anticancer activity of the triterpene glycosides from sea star *S. pacificus* is still unclear. Therefore, investigating the molecular mechanisms underlying the anticancer activity of pacificusoside C and cucumariosides C1 and C2 is of high interest.

## Results

### Cytotoxic activity of isolated triterpene glycosides in human prostate cancer cells

**Fig. 1:**
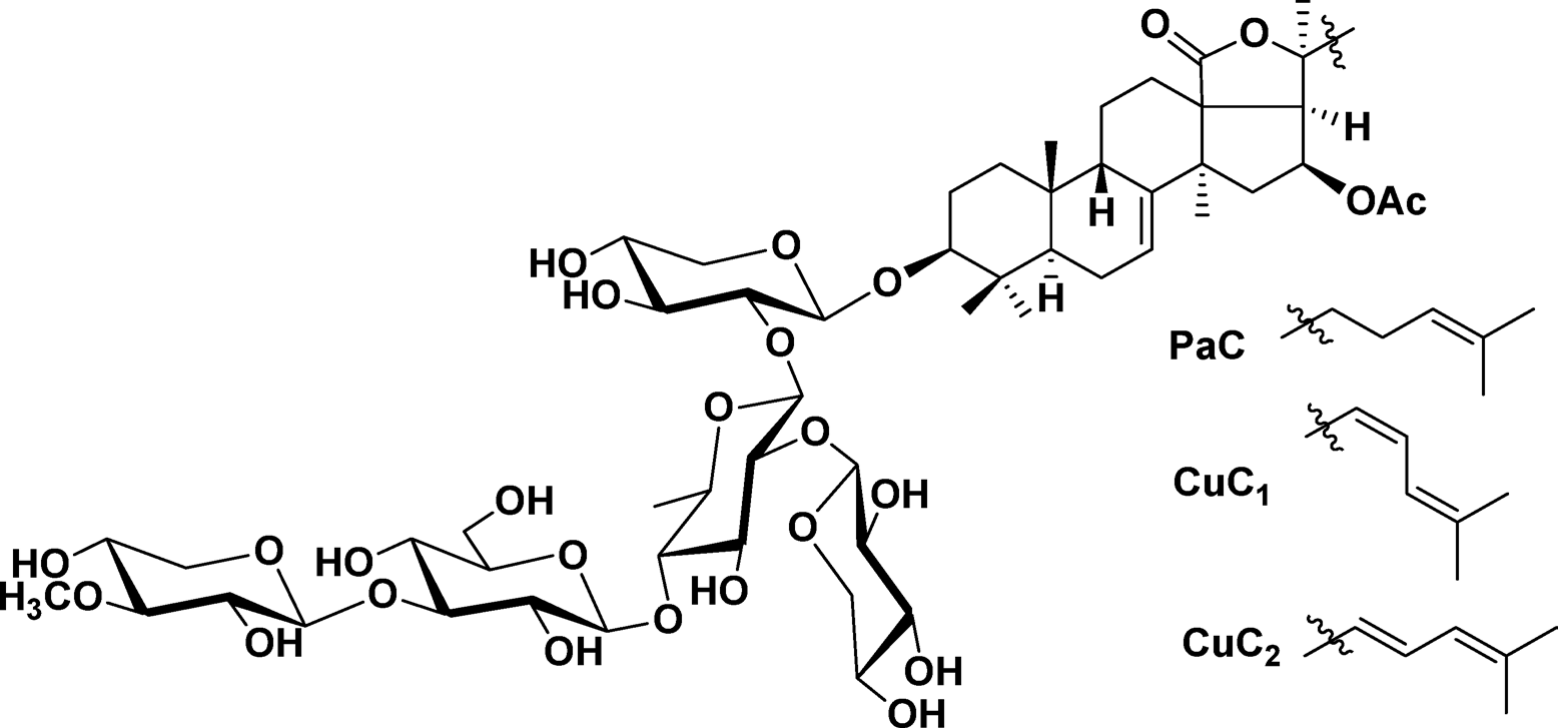
Chemical structures of pacificusoside C (PaC), cucumariosides C_1_ (CuC_1_), and C_2_ (CuC_2_) isolated from sea star *S. pacificus*.

The triterpene glycosides pacificusoside C (PaC), cucumarioside C_1_ (CuC_1_), and cucumarioside C_2_ (CuC_2_) were isolated from the sea star *S. pacificus* and purified as previously described (Fig. 1)^15^. The anticancer properties of the isolated compounds were evaluated as their cytotoxic activity in a series of human prostate cancer and non-cancer cell lines using an MTT assay. Specifically, we tested their effects on prostate cancer cell lines PC3, PC3-DR, DU145, VCaP, 22Rv1, and LNCaP, as well as on non-malignant human cell lines PNT2, RWPE-1, MRC-9, and HEK293T^15^. Among these, LNCaP represents a hormone-sensitive cell line, whereas the other cancer cell lines exhibit resistance to androgen synthesis inhibitor abiraterone and androgen receptor (AR)-inhibitors such as enzalutamide. In 22Rv1 and VCaP cells, this resistance is mediated by the expression of AR-V7 (AR splice variant 7), which lacks an androgen-binding site, leading to constitutive activation of the AR pathway^30,31^. In contrast, PC3, PC3-DR, and DU145 cells do not express AR and proliferate independently of androgens^30^. The PC3-DR subline represents docetaxel-resistant cells derived by gradually increasing exposure of PC3 cells to this docetaxel^32^.

The investigated compounds demonstrated cytotoxic effects in all evaluated cancer cell lines at low micro- and nanomolar concentrations, with the most pronounced activity observed in 22Rv1 and VCaP cells (Table 1). Interestingly, hormone-refractory PC3 and DU145 cells exhibited sensitivity comparable to hormone-sensitive LNCaP cells (Table 1). Docetaxel is a chemotherapeutic agent often used for treatment of castration-resistant prostate cancer^3,4^. Remarkably, PC3-DR cells, that demonstrated 35-fold reduced sensitivity to docetaxel compared to parental PC3 cells, displayed only a minor (2–to–threefold higher compared to PC3 cells) resistance to the triterpene glycosides (Table 1). This suggests a minimal cross-resistance between the tested compounds and docetaxel (Table 1). Furthermore, the cytotoxic activity of the natural compounds was comparable to the activity of cisplatin, a drug that is commonly used for the treatmen of AVPC^33^ (Table 1). We further analyzed activity of the isolated marine triterpene glycosides cancer cells versus non-cancer cells. In our in vitro experiments, the investigated triterpene glycosides were slightly more cytotoxic to non-cancer cells and therefore did not exhibit any pronounced selectivity to cancer cells. The selectivity index of CuC_1_ was comparable to that of cisplatin (0.7 and 0.88, respectively), whereas PaC and CuC_2_ had lower selectivity indexes (Table 1).

**Table 1:** Cytotoxicity of PaC, CuC_1_, and CuC_2_ observed in human prostate cancer and human non-cancer cell lines.

	IC_50_ ([μM]* or [nM]**)										SI
	Cancer cells						Non-cancer cells				
	PC3	PC3-DR	DU145	22Rv1	VCaP	LNCaP	PNT2	RWPE-1	HEK293T	MRC-9	
PaC*	5.68 ± 0.32	13.60 ± 4.22	15.50 ± 2.13	1.16 ± 0.07	1.86 ± 0.12	8.24 ± 1.07	3.03 ± 0.24	4.50 ± 0.23	2.85 ± 0.13	2.11 ± 0.27	0.43
CuC_1_*	1.59 ± 0.12	5.69 ± 0.78	3.24 ± 0.34	0.246 ± 0.017	0.472 ± 0.065	3.0 ± 0.54	0.754 ± 0.058	0.554 ± 0.105	0.66 ± 0.05	0.959 ± 0.248	0.7
CuC_2_*	2.21 ± 0.14	6.2 ± 1.47	2.69 ± 0.19	0.623 ± 0.035	0.487 ± 0.042	2.18 ± 0.19	1.270 ± 0.054	0.897 ± 0.066	1.59 ± 0.10	0.799 ± 0.062	0.48
Doce**	11.40 ± 3.71	388 ± 49.5	2.20 ± 0.61	0.604 ± 0.122	0.225 ± 0.215	5.06 ± 0.58	> 500	0.423 ± 0.225	11.49 ± 3.22	> 500	10.3
Cis*	34.60 ± 6.62	6.89 ± 2.29	1.32 ± 0.31	0.993 ± 0.2	5.02 ± 2.73	2.75 ± 0.39	9.41 ± 1.82	9.02 ± 1.52	6.44 ± 0.65	6.75 ± 2.15	0.88

Cells were treated with the investigated compounds for 48 h, viability was evaluated using MTT assay, and IC_50_ values were calculated using GraphPad Prism software. Docetaxel (Doce) and cisplatin (Cis) were used as positive controls. The values are presented as mean ± standard deviation in μM (PaC, CuC_1_, CuC_2_, and Cis) or (**) in nM (Doce). Selectivity index (SI) was calculated as [mean IC_50_ in non-cancer PNT2, RWPE-1, HEK293T, and MRC-9 cells] / [mean IC_50_ in cancer PC3, 22Rv1, DU145, VCaP, LNCaP cells]. The experiment was performed in triplicates (number of biological replicates *n* = 3). R2 values (goodness-of-fit) for the dose–response curves used to calculate IC₅₀ values are presented in the Supplementary Table S1.

Thus, CuC_1_ was the most active compound among the tested triterpene glycosides. Therefore, it was chosen for further experiments as the most promising molecule. To tackle the mode of action of CuC_1_ we chose highly aggressive hormone- and docetaxel-refractory PC3-DR cells, as well as 22Rv1 cells, which represent a hormone-refractory AR-V7-positive prostate cancer subtype.

### CuC_***1***_ affects cancer cells independently of their P-glycoprotein status

Next, we investigated the low-grade cross-resistance to CuC_1_, observed in the docetaxel-resistant PC3-DR cell model (Table 1). A major contributor to docetaxel resistance is P-glycoprotein (also known as P-gp, MDR1), known to be overexpressed in PC3-DR cells^34^. P-glycoprotein is a molecular efflux pump that actively expels various xenobiotic agents, including docetaxel, from cancer cells^35^. Increased P-gp expression correlates with an increased IC_50_ for docetaxel, signifying enhanced drug resistance^36^. The observation that PC3 and PC3-DR cells exhibit similar sensitivity to the investigated compounds suggests that these drugs are not P-gp substrates (Table 1). To further validate this, we evaluated the effect of the isolated triterpene glycosides on P-gp activity using a calcein-AM exclusion assay. In this assay, the non-fluorescent calcein-AM passively diffuses into cells, where it is hydrolyzed by cellular esterases into fluorescent calcein. Both calcein and its precursor are P-gp substrates and are actively transported out of P-gp-highly expressing cells (e.g. PC3-DR cells), which results in reduced intracellular fluorescence. P-gp inhibitors, such as tariquidar, block P-gp function, leading to calcein accumulation and an increase in fluorescence, which therefore can be used as a reliable indicator of reduced P-gp activity^37^. A similar fluorescence increase is observed in the presence of docetaxel due to its competitive binding to the P-gp transporter alongside calcein^38^.

**Fig. 2:**
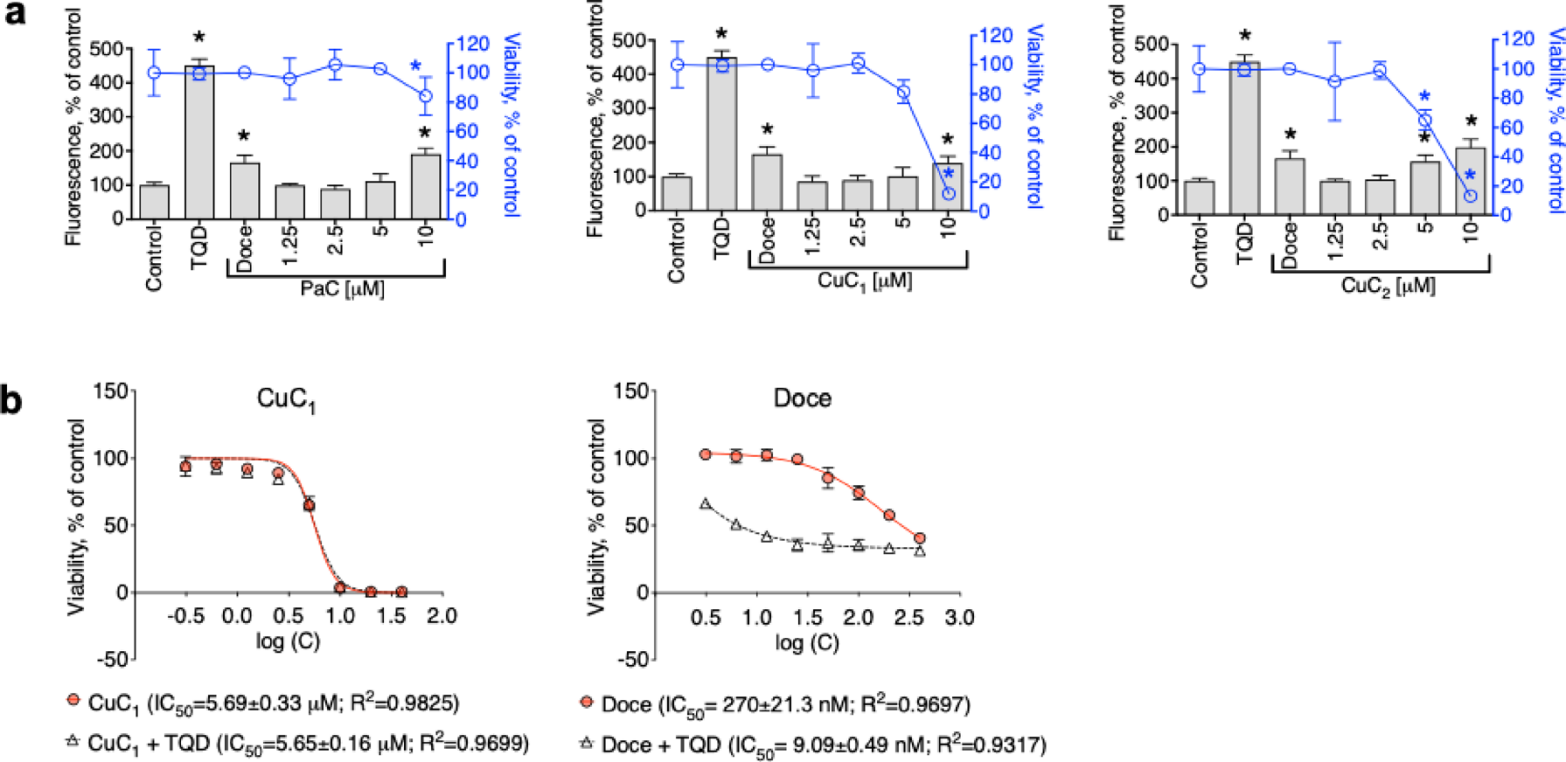
Effect of the triterpene glycosides on P-gp activity in PC3-DR cells. (**a**), The effect of the investigated compounds on P-gp activity (grey bars) and cell viability (blue graph). PC3-DR cells were treated with the drugs for 30 min, and P-gp activity was evaluated using the calcein-AM assay, while cellular viability was determined via the MTT test in the same experimental conditions. P-gp inhibitor tariquidar (TQD, 50 nM) and a P-gp substrate docetaxel (Doce, 100 nM) were used as positive controls. Statistically significant difference from control was determined using a one-way ANOVA test, *p < 0.05. Number of biological replicates *n* = 3. (**b**), The effect of TQD on cytotoxic activity of CuC_1_ and Doce. PC3-DR cells were pre-treated with TQD (50 nM) for 1 h and then co-treated with CuC_1_ or Doce for another 48 h. Viability was evaluated using MTT assay, and IC_50_s were calculated using GraphPad Prism software. Number of biological replicates *n* = 3.

In our experiments, incubation of PC3-DR cells with CuC_1_ at non-cytotoxic concentrations did not result in any significant changes in calcein-AM efflux, whereas docetaxel- or tariquidar-treated cells expectedly affected P-gp activity (Fig. 2a) via the mechanisms discussed above. At the same time, an increase of intracellular fluorescence was observed at high cytotoxic concentrations of CuC_1_, which normally indicates an interrupted calcein excretion out of the cells due to the disruption of cellular membrane integrity by a cytotoxic agent. Notably, similar effects were also observed for PaC and CuC_2_ (Fig. 2a). This data suggests the investigated glycosides to be neither P-gp substrates nor P-gp inhibitors. In line with this observation, co-treatment of cells with tariquidar did not alter cytotoxicity of CuC_1_ in PC3-DR cells, whereas the activity of docetaxel was expectedly dramatically affected resulting in 30-fold decrease of IC_50_ (Fig. 2b)^39^. Taken together, cytotoxicity of CuC_1_ and related compounds was exerted independently of P-gp status, and therefore the compound was active in P-gp-overexpressing cancer cells.

### Effect of CuC_***1***_ on serine/threonine kinases in prostate cancer cells

Protein kinases regulate cellular processes through phosphorylation, with serine/threonine kinases (STKs) playing critical roles in cell death and survival. Several FDA-approved anticancer agents, including palbociclib, trametinib, and selumetinib target STKs^40^. Therefore, we employed functional kinome profiling using the PamTechnology platform (http://www.pamgene.com) to identify the STKs affected in prostate cancer cells treated with CuC_1_. This approach utilizes exposure of a set of immobilized peptide STK substrates to cell lysates, and changes in peptide phosphorylation patterns indicate alterations in the activity of respective kinases.

**Fig. 3:**
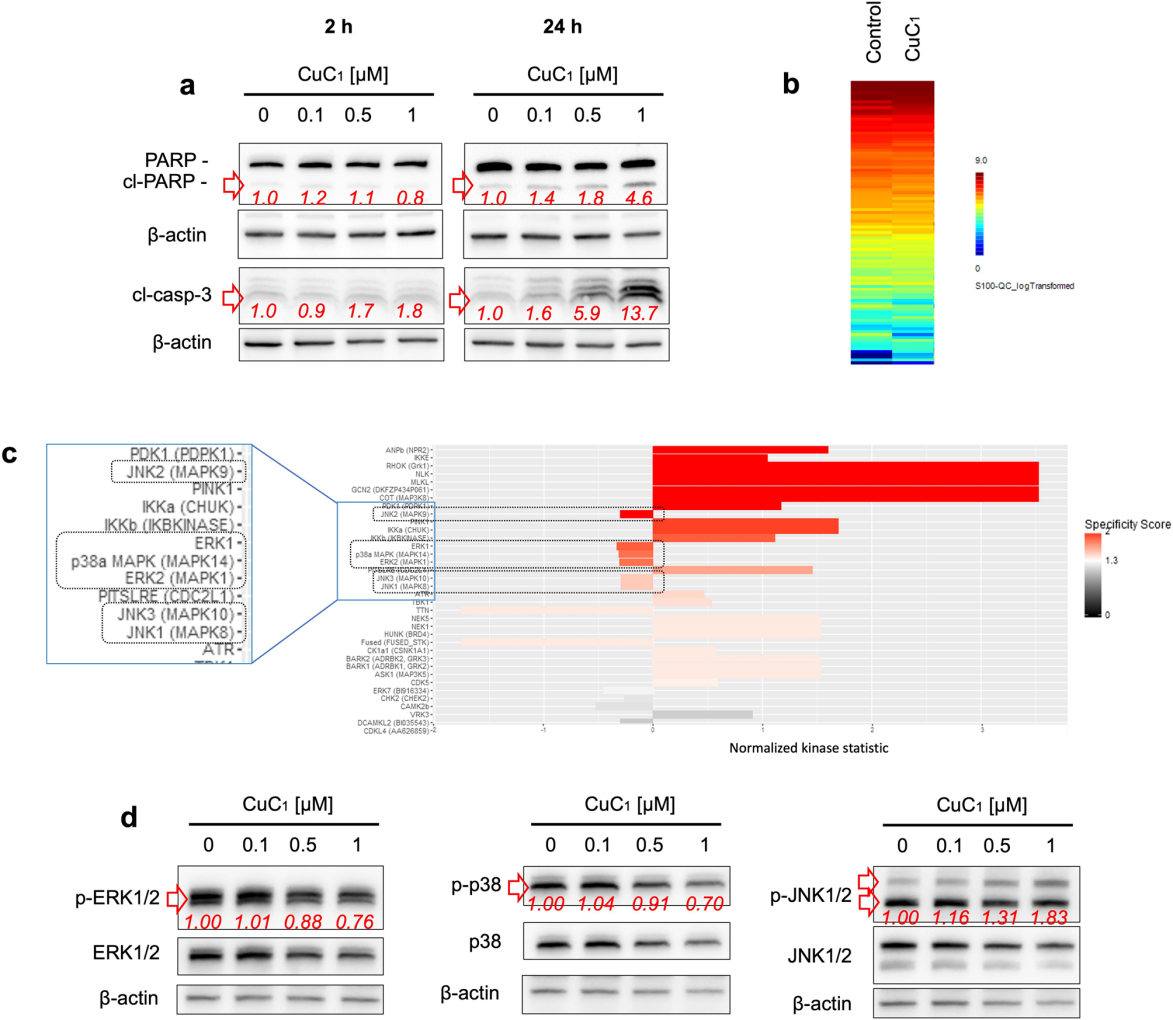
Functional kinome profiling of STKs (serine/threonine kinases) in 22Rv1 cells and validation of results. (**a**) Expression of apoptotic markers. The cells were treated with CuC_1_ for the indicated time, and the expression of proteins was analyzed by Western blotting. (**b, c**) Functional kinome profiling. A heatmap displaying log2-transformed phosphorylation levels of 99 analyzed STK substrate peptides (**b**) and upstream kinase analysis, which predicted kinases affected by CuC_1_ (**c**). Values of the normalized kinase statistics represented by bars, where log2 < 0 indicates decreased kinase activity and log2 > 0 denotes increased activity. Statistically significant changes having a specificity score log2 > 1.3 are highlighted by red bars. Number of biological replicates *n* = 3. (**d**), Western blot analysis of MAP kinases. 22Rv1 cells were treated with CuC_1_ for 2 h and the MAPK expression was analyzed by Western blotting. β-actin was used as a loading control. The band intensity of the protein of interest was quantified using Image Lab software and normalized to the band intensity β-actin (**a**), or to the band intensity of non-phospho MAPK (**d**), and is indicated by red numbers. Full-size blots are presented in Supplementary Fig. S2.

We intended to identify the primary/early effects of the drug. Therefore, to minimize secondary events resulting from cell death, short-term drug exposure was applied. For this assay, we used 22Rv1 cancer cells as a model, as this cell line was most sensitive to CuC_1_ (Table 1). We demonstrated that treatment with CuC_1_ for 2 h at twice the IC_50_ concentration (0.5 μM, selected to maximize the effect on kinome at a short exposure time) did not induce any signs of cell death in 22Rv1 cells. However, after 24 h of treatment, apoptotic markers such as cleaved PARP and caspase-3 were detected (Fig. 3a). Thus, short-term treatment regime (0.5 μM, 2 h) was further used to identify the affected STKs. The results of kinome profiling analysis were represented as log2-transformed peptide signal intensities of treated samples compared to control (Fig. 3b). Further upstream kinase analysis suggested alteration of the activity of multiple kinases following CuC_1_ treatment. This included activation of kinases involved in stress response and cell survival pathways (IKKα, IKKβ, IKKε), necroptosis (MLKL), metabolic regulation (GCN2, PDK1), cytoskeletal dynamics and migration (RHOK), mitophagy (PINK1), apoptosis and cell cycle regulation (PITSLRE), inflammatory and immune modulation (COT) (Fig. 3c). Notably, few STKs identified as MAP kinases, namely, p38, ERK1/2, and JNK1/2 were predicted to be significantly inhibited by the drug (Fig. 3c).

The vast majority of modern anticancer drugs used in the clinic are kinase inhibitors^41^. Hence, we focused on STKs predicted to be inhibited by CuC_1_. To validate the findings of functional kinome profiling, Western blot analysis was employed. Thus, we could confirm an inhibition of active (phosphorylated) forms of ERK1/2 and p38, but not JNK1/2, in cells exposed to the drug for 2 h (Fig. 3d). This was confirmed by analysis of phosphor-MAPK / MAPK ratio (Fig. 3d). Both ERK1/2 and p38 kinases were previously associated with tumor growth, invasion as well as drug resistance^42,43^. Notably, saponin from bark of the *Quillaja saponaria* tree, which was used as a reference compound, had a distinct effect on the kinome of cells and induced an activation of ERK1/2 kinase in similar experimental conditions, whereas no significant effect on p38 kinase was registered (Supplementary Fig. S1).

### CuC_1_ potentiates cytotoxicity of taxanes and platinum agents in prostate cancer cells

**Fig. 4:**
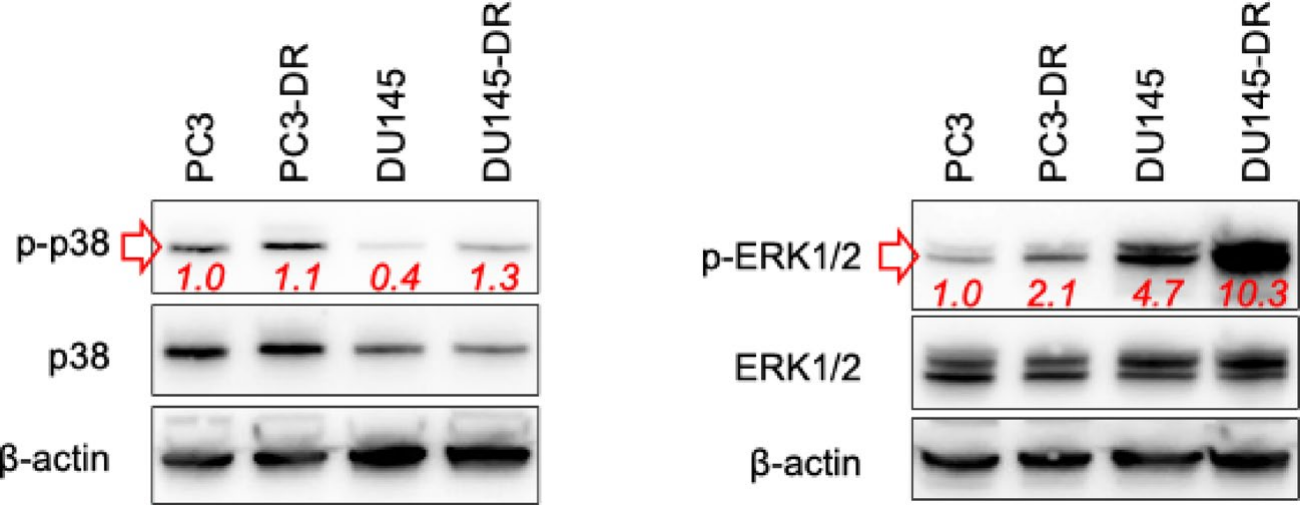
Expression of p38 and ERK1/2 in the taxane-resistant and taxane-sensitive prostate cancer cells. The protein expression was analyzed by Western blotting. β-actin was used as a loading control. The band intensity of the protein of interest was quantified using Image Lab software and normalized to the band intensity of non-phospho MAPK, and is indicated by red numbers. Full-size blots are presented in Supplementary Fig. S2.

Next, we evaluated the potential clinical relevance of CuC_1_ in combination with several approved chemotherapeutics. Taxanes are commonly used in the treatment of advanced prostate cancer, whereas platinum-based therapies demonstrate significant efficacy in patients with DNA repair deficiencies or AVPC^33^. Moreover, cisplatin (Cis) and carboplatin (Carbo) were reported to activate all three MAPKs, whereas docetaxel (Doce) and cabazitaxel (Caba) activated p38 and JNK1/2 while inhibiting ERK1/2 in prostate cancer cells^44^. Additionally, persistent ERK1/2 signaling was identified to mediate taxane-resistance in prostate cancer^45^. Thus, we first showed that p38 and ERK1/2 kinases are overactivated in the taxane-resistant PC3-DR and DU145-DR prostate cancer cells compared to their drug-sensitive counterparts (Fig. 4). Then, we investigated the effects of CuC_1_ in combination with Cis, Carbo, Doce, and Caba in prostate cancer cells. Accordingly, we used docetaxel- and hormone-resistant PC3-DR cells as well as hormone-resistant 22Rv1 cells.

**Fig. 5:**
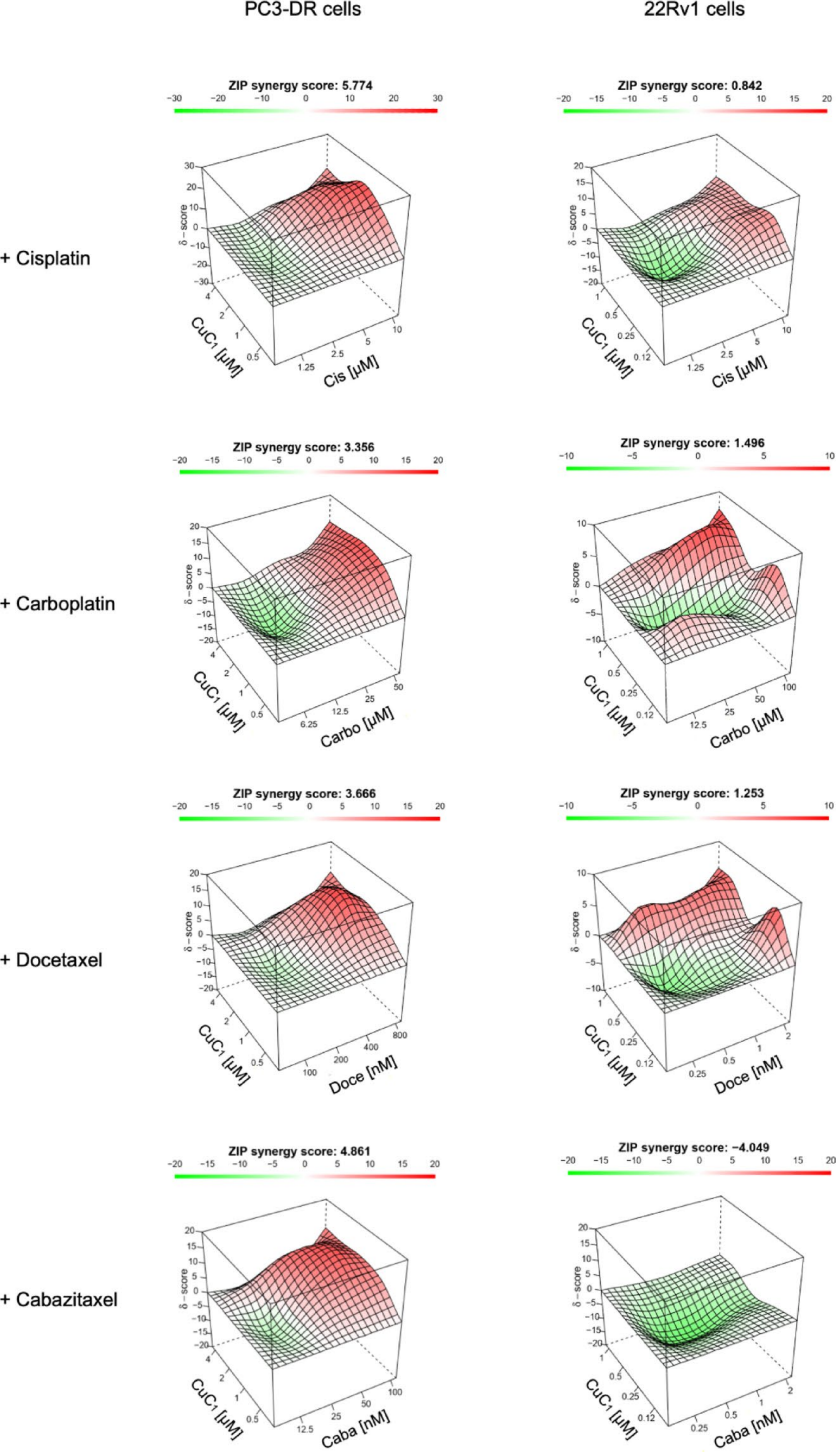
Effects of CuC_1_ in combination with taxanes and platinum agents. PC3-DR and 22Rv1 cells were co-treated with cisplatin (Cis), carboplatin (Carbo), docetaxel (Doce), or cabazitaxel (Caba) in combination with CuC_1_ at the indicated concentrations for 48 h. Cellular viability was evaluated using the MTT assay (number of biological replicates *n* = 3.) and is represented in the Supplementary Table S2. The cytotoxic effects were further analyzed, and 3D heat maps were constructed using SynergyFinder 3.0 software. The drug effects in combination (synergistic, additive, or antagonistic) were analyzed and visualized using the ZIP algorithm and are indicated by δ-score. δ > 10: Synergistic interactions (dark red area); − 10 > δ > 10: additive effect; δ < − 10: Antagonistic interactions (dark green area). A ZIP synergy score represents a mean δ overall the concentration range used.

The effects of CuC_1_ in combination with platinum agents and taxanes were similar in both PC3-DR and 22Rv1 cells. Specifically, a synergistic effect was detected when higher doses of Cis, Carbo, Doce, and Caba were combined with CuC_1_, whereas an additive effect was observed at lower concentrations of these chemotherapeutics in their combination with CuC_1_ (Fig. 5). In 22Rv1 cells the overall effects of these combinations were additive, which was indicated by a *δ*-score being in the range of − 10 < δ < 10, while for PC3-DR cells the areas with δ > 10 were detected indicating a true synergism (Fig. 5). However, it should be noted that for combination CuC_1_ plus Caba in 22Rv1 cells, the lowest detected δ value was − 10.7, indicating a minimal antagonistic effect at the defined concentration range (Fig. 5).

## Discussion

Marine triterpene glycosides possess various biological activities, including anticancer activity. In this study, we evaluated the effects of three marine triterpene glycosides pacificusoside C, cucumariosides C_1_ and C_2_ (PaC, CuC_1_, CuC_2_) in prostate cancer models in vitro. While the compounds possessed a selectivity comparable to the approved anticancer medication cisplatin, its activity in cancer cells was not affected by an overexpression of P-gp—a major mechanism of multidrug resistance of various cancer entities. Therefore, the minor cross-resistance observed in the docetaxel-resistant PC3-DR cells is related to factors other than P-gp status of the cells. Among possible factors, there are modulations of cellular metabolism, tubulin dynamics, altered cellular membrane composition, different proliferative rates of the cells, or activation of general pro-survival and antiapoptotic pathways^5^. Moreover, taxane-resistant cells were shown to have an altered metabolism of cholesterol, one of the primary targets of saponins, resulting in an altered cholesterol content in the cellular membrane^46^. Such alterations may result in reduced sensitivity to saponin-like compounds^28^.

To the best of our knowledge, the current research is the very first study of the effect of triterpene glycosides on the kinome of cancer cells. Kinome profiling revealed a possible induction of multiple kinases in the cells treated with CuC_1_. Thus, predicted IKKα, IKKβ, and IKKε kinases are a part of the NF-κB signaling pathway, which regulates cell survival, inflammation, and stress responses. Activation of this pathway promotes either cell survival (in the vast majority of cases) or apoptosis, depending on the biological context^47^. Moreover, in prostate cancer, IKKβ is known to mediate cancer cell stemness and epithelial-to-mesenchymal transition, and therefore, potentially contributes to tumor progression and drug resistance^48^. On the contrary, activation of MLKL, an important mediator of necroptosis, proposes this type of programmed cell death is also involved in the cytotoxic action of CuC_1_ and related drugs^49^. Activation of PITSLRE kinase suggests promotion of apoptotic pathways or disruption cell cycle progression in the treated cancer cells^50^, which was in line with the detected upregulation of other apoptotic markers (Fig. 3a).

The predicted activation of GCN2 and PDK1 further proposes that CuC_1_ affects cellular metabolism^51,52^. Additionally, PDK1 is a mediator of the PI3K/AKT pathway, and the activation of this axis may contribute to the resistance of the cancer cells^52^. Rho kinase is known to regulate cytoskeletal dynamics and increase or suppress motility, depending on the cellular context^53^, while PINK1 is involved in mitophagy and is a part of a mitochondria quality control system^54^. COT (MAP3K8) kinase is involved in inflammatory signaling, and its modulation could affect the tumor microenvironment or immune responses^55^. However, the activation of the above-mentioned kinases should further be validated using alternative methods.

Taken together, the results of the functional kinome profiling suggest CuC_1_ to simultaneously modulate several processes, including metabolism, survival, mitochondrial quality control, and cytoskeletal dynamics. The cells also seem to activate prosurvival pathways, including NF-κB and PI3K/AKT signaling, that may partially mitigate the effect of the drug. This hypothesis awaits further validation in upcoming studies.

A very interesting finding was CuC_1_-mediated inhibition of ERK1/2 and p38 MAP kinases, that was the first predicted by functional kinome profiling and further validated by Western blotting. Recently, we reported a chemopreventive activity pacificusoside D and cucumarioside D, triterpene glycosides structurally closely related to CuC_1_, in the JB6 Cl41 cells model^16^. In this model, the JB6 Cl41 cells were exposed to epidermal growth factor (EGF) or a chemical carcinogen 12-*O*-tetradecanoilphorbol-13-acetate (TPA) to induce the malignant transformation of the cells^56^. Previously, it was shown that activation of MAPKs is a critical step of the malignant transformation event in JB6 Cl41 model and that MAPK inhibition suppresses this process^56^. Thus, the inhibition of ERK1/2 and p38 MAPKs reported in the current research may explain the cancer-preventive activity of CuC_1_ and related compounds. As the activity of multiple kinases was predicted to be affected in CuC_1_-treated cells (Fig. 3c), it is unlikely that CuC_1_ acts as a specific molecular inhibitor of p38 and ERK1/2 kinases. Instead, we propose that the observed inhibition of p38 and ERK1/2 activity in prostate cancer cells is a secondary effect of CuC_1_ treatment, potentially resulting from various biological mechanisms that warrant further investigation.

Finally, we showed that CuC_1_ potentiates the cytotoxic effects of cisplatin, carboplatin, docetaxel, and cabazitaxel in PC3-DR and 22Rv1 cells. This observation can be potentially explained by the ability of CuC_1_ to inhibit ERK1/2 and p38 MAPK pathways, which are known to be over-activated in the docetaxel-resistant prostate cancer cells and contribute to their drug resistance^57^. This makes CuC_1_ a potential combinational partner for the above-mentioned chemotherapeutics, however, further research is required here to analyze the combinational treatment options in more detail, including an in vivo safety and efficacy profiles. Additionally, our data contributes to the understanding of the anti-cancer properties of some triterpene glycosides produced by marine invertebrates that are used in Traditional Chinese Medicine for cancer treatment^29^.

## Conclusion

In summary, the marine triterpene glycosides pacificusoside C, cucumariosides C_1_ and C_2_ were shown to be cytotoxic in human prostate cancer cells bearing various levels of drug resistance. The compounds were active in docetaxel-resistant prostate cancer cells, were neither P-glycoprotein inhibitors nor substrates, and exerted their cytotoxicity independently of the P-glycoprotein status. Additionally, cucumarioside C_1_ potentiates cytotoxic effects cisplatin, carboplatin, docetaxel or cabazitaxel in the docetaxel-resistant cells. Cucumarioside C_1_ was revealed to activate kinases involved in various cellular processes, such as metabolic regulation, cytoskeletal dynamics and migration, cell cycle regulation, apoptosis, necroptosis, survival, mitophagy, inflammatory and immune modulation. Moreover, we revealed and validated a CuC1-mediated inhibition of p38 and ERK1/2 MAP kinases in prostate cancer cells. This phenomenon may explain some anticancer effects previously reported for cucumarioside C_1_ and related compounds. To the best of our knowledge, this is the very first study reporting the effect of triterpene glycosides on the kinome of cancer cells.

## Materials and methods

### Marine natural compounds

Pacificusoside C, cucumariosides C_1_ and C_2_ were isolated from the sea star *Solaster pacificus* as previously described^15^. Specimens of *S. pacificus* were collected near Iturup Island (Sea of Okhotsk) by scuba diving in August 2012^15^. The purity of compounds was confirmed by ^1^H NMR, TLC, and ESI mass-spectrometry (Supplementary Fig. S3-S15). For the biological experiments, the stock solutions of compounds in 100% DMSO (vehicle) were used.

### Reagents and antibodies

The following reagents were used: MTT (3-(4,5-dimethylthiazol-2-yl)-2,5-diphenyltetrazolium bromide) (Sigma, Taufkirchen, Germany); tariquidar (P-glycoprotein inhibitor) (MedChemExpress, Monmouth Junction, NJ, USA); calcein-AM (BIOZOL, Eching, Germany); MTS viability assay kit (CellTiter 96 Aqueous One Solution Reagent, Promega, Madison, WI, USA); cisplatin, carboplatin, docetaxel, and cabazitaxel (Pharmacy of the University Hospital Hamburg-Eppendorf, Hamburg, Germany).

The following primary and secondary antibodies were used: Rabbit anti-cleaved Caspase-3 (mAb, #9664, 1:1000, Cell Signaling), mouse anti-ERK1/2 (mAb, #9107, 1:2000, Cell Signaling), rabbit anti-JNK1/2 (mAb, #9258, 1:1000, Cell Signaling), rabbit anti-p38 (mAb, #9212, 1:1000, Cell Signaling), rabbit anti-PARP (pAb, #9542, 1:1000, Cell Signaling), rabbit anti-phospho-ERK1/2 (mAb, #4377, 1:1000, Cell Signaling), rabbit anti-phospho-JNK1/2 (mAb, #4668, 1:1000, Cell Signaling), rabbit anti-phospho-p38 (mAb, #4511, 1:1000, Cell Signaling), goat anti-rabbit IgG-HRP (#7074, 1:5000, Cell Signaling), mouse anti-α-tubulin (mAb, T5168, 1:5000, Sigma-Aldrich), goat anti-β-actin-HRP (pAb, sc-1616, 1:10,000, Santa Cruz), sheep anti-mouse IgG-HRP (NXA931, 1:10,000, GE Healthcare).

### Cell lines and culture conditions

The cell lines were purchased from ATCC (Manassas, VA, USA) or ECACC (Salisbury, UK). The following cell lines were used: Human prostate cancer cells LNCaP (ATCC; RRID:CVCL_0395), VCaP (ECACC; RRID:CVCL_2235), 22Rv1 (ATCC; RRID:CVCL_1045), PC3 (ATCC; RRID:CVCL_0035), and DU145 (ATCC; RRID:CVCL_0105); human prostate non-cancer cells PNT2 (ATCC; RRID:CVCL_2164) and RWPE-01 (ATCC; RRID:CVCL_3791). Human non-cancer embryonic kidney cells HEK293T (ECACC; RRID:CVCL_0063) and human fibroblast cells MRC-9 (ECACC; RRID:CVCL_2629). The docetaxel-resistant cells PC3-DR were generated by long-term treatment of human prostate cancer PC3 cells with sublethal concentrations of docetaxel as previously described^32^ and were provided by Prof. Z. Culig (Innsbruck Medical University, Austria). All cell lines were recently authenticated by a commercial service (Multiplexion GmbH, Heidelberg, Germany) and were cultured as previously described^58,59^. Cells were kept in culture for a maximum of three months and were routinely examined for bacterial, fungal, and mycoplasma infections.

### MTT assay

Cell viability was measured using the MTT assay. Cells (6 × 10^3^ cells/well in 100 μL/well of media) were plated in 96-well plates. The plates were incubated overnight, and the medium was replaced with fresh medium containing drugs at the indicated concentrations. The plates were further incubated for 48 h, unless otherwise stated, the solution of MTT reagent in PBS was added to each well, and cells were incubated for another 2–4 h to form formazan crystals. Then the media was removed and the plates were dried overnight. 50 μL of DMSO was added to each well to dissolve the formazan crystals. The absorbance of each well was measured using an Infinite F200PRO reader (TECAN, Männedorf, Switzerland). The cell viability was estimated as an inhibition concentration (IC_50_), which was calculated using GraphPad Prism software v.9.1.1 (GraphPad Software, San Diego, CA, USA). IC₅₀ values were determined by fitting the data to a nonlinear regression model (log[inhibitor] versus normalized response—variable slope). R^2^ values (goodness-of-fit) for the dose–response curves used to calculate IC₅₀ values are represented on the respective figures and in the Supplementary Table S1. A selectivity index (SI) was calculated as: SI = [(IC_50_(PNT2) + IC_50_(RWPE-1) + IC_50_(HEK293T) + IC_50_(MRC-9))/4]/[(IC_50_(PC3) + IC_50_(22Rv1) + IC_50_(DU145) + IC_50_(VCaP) + IC_50_(LNCaP))/5]. For calculation of SI the IC_50_ values for Doce in PNT2 and MRC-9 cells were assumed to be 500 nM.

### Examination of drug effects in combination

The effect of combinational treatment with established chemotherapies was evaluated as previously reported^54^. In brief, cells were exposed to individual drugs of their combinations for 48 h, and the viability was measured by MTT assay and was then analyzed using SynergyFinder 3.0 software (https://synergyfinder.fimm.fi) and the ZIP score algorithm^60^. Statistical analysis was conducted automatically by the software using its inbuilt algorythms based on the input data type and size. The effects of drug combinations (synergistic, additive, or antagonistic) were assigned using a *δ*-score, where δ > 10 indicated synergistic interactions (dark red area), − 10 > δ > 10 refers to additive effect, and δ < − 10 suggests antagonistic interactions (dark green area). The raw cell viability data used for synergy analysis presented as % of effect (0 refers to 0% of dead cells compared to the control, i.e. no cytotoxicity effect; 100 refers to 100% dead cells compared to the control) can be found in the Supplementary Table S2.

### P-gp activity assay

The evaluation of the activity of P-glycoprotein (P-gp) was performed using calcein-AM assay as described previously^34^. P-gp-overexpressing PC3-DR cells (10 × 10^3^ cells/well in 100 µL/well of media) were seeded in a black 96-well plate with clear flat bottoms. Plates were incubated overnight, and the medium was replaced with 50 µL of PBS containing the tested drug at the indicated concentrations and incubated for 30 min. The calcein-AM solution in PBS (50 µL) was added to each well to achieve a final calcein-AM concentration of 1 µM. The plates were incubated for 15 min, and the green calcein fluorescence was recorded using an Infinite F200PRO plate reader (TECAN). The viability of cells treated in the same experimental settings was evaluated using the MTS assay as described previously^34^.

### Functional kinome profiling

Functional kinome profiling was performed as described previously^61^. The PamStation®12 system and STK-PamChip® array (PamGene International, ´s-Hertogenbosch, The Netherlands) were used. The array is designed to analyze the activity of serine-/threonine kinases (STKs), each chip contains 140 peptide phospho-sites that are analogs for the substrates of respective STKs. The proteins were extracted from the 22Rv1 cells treated with CuC_1_ (0.5 µM, 2 h) or vehicle-treated cells using M-PER Mammalian Extraction Buffer supplemented with protease and phosphatase inhibitor cocktails (Pierce, Waltham, Massachusetts, USA). The cell lysates were mixed with ATP and applied on the chip according to the manufacturer’s protocol. The phosphorylation of specific peptide sequences was then detected using primary anti-phospho-Ser/Thr and secondary immunoglobulin-FITC antibodies (PamGene International). The signals were recorded using a dedicated camera and Evolve software v. 1.0 (PamGene International). The quality of the signals was controlled, and the signal intensities were log2-transformed and analyzed using the BioNavigator software v. 6.0 (PamGene International).

### Western blotting

Western blotting was performed as previously described^62^. In brief, 22Rv1 cells were seeded in Petri dishes (1 × 10^6^ cells/dish in 10 mL/dish of media), incubated overnight, and treated with the drug for a specific time. Cells were harvested by scratching, washed with cold PBS, and lysed with RIPA buffer supplemented with protease and phosphatase inhibitor cocktails. Protein lysates were separated using precast Mini-PROTEAN® TGX Stain-FreeTM gels (Bio-Rad, Hercules, CA, USA) and SDS-PAGE. Proteins were transferred on a PVDF membrane and detected using specified primary and secondary antibodies. The protein signals were visualized and detected using the ECL chemiluminescence system (Thermo Scientific, Rockford, IL, USA). The original blot images were cropped, and the figures were composed using MS PowerPoint software v. 2310, build 16924.20150 (Microsoft Inc., Redmond, WA, USA). The signal (band) intensity was quantified using Image Lab v. 6.1.0. build 7 software (Bio-Rad Laboratories, Hercules, CA, USA) and normalized to loading control (for non-phospho proteins), or normalized to signal intensity of non-phospho proteins (for phospho proteins). The original uncropped images are available in Supplementary Fig. S2.

### Data and statistical analysis

Statistical analysis was performed using GraphPad Prism software v.9.1.1 (GraphPad Prism software Inc., La Jolla, CA, USA). The data are represented as mean ± standard deviation (SD). For the comparison of multiple groups, one-way ANOVA combined with Dunnett’s post-hoc tests was used. All experiments were carried out in triplicate (*n* = 3, biological replicates), unless otherwise stated. The difference between the groups was considered to be statistically significant and indicated with an asterisk (*) if *p* < 0.05.

## Electronic supplementary material

Below is the link to the electronic supplementary material.

Supplementary Material 1

## Data Availability

The original data are available in the Supplementary as well as from the corresponding author on a reasonable request.
