# Supplementary material for: Anticancer activity of triterpene glycosides from the sea star Solaster pacificus
Source: Sci Rep. 2025 Aug 4;15:28410. doi: 10.1038/s41598-025-12914-7 (PMC12322260; doi:10.1038/s41598-025-12914-7)

Supplementary information

Anticancer activity of triterpene glycosides from the sea star *Solaster pacificus*

Sergey A. Dyshlovoy[1,*], Jessica Hauschild[1], Malte Kriegs[2,3], Konstantin Hoffer[2,3], Olga Y. Burenina[4], Nadja Strewinsky[1], Timofey V. Malyarenko[5,6], Alla A. Kicha[5], Natalia V. Ivanchina[5], Valentin A. Stonik[5], Markus Graefen[7], Carsten Bokemeyer[1], Gunhild von Amsberg[1,7]

1 Laboratory of Experimental Oncology, Department of Oncology, Hematology and Bone Marrow Transplantation with Section Pneumology, Hubertus Wald-Tumorzentrum, University Medical Center Hamburg-Eppendorf, Martinistrasse 52, 20251 Hamburg, Germany; s.dyshlovoy@uke.de (S.A.D.), j.hauschild@uke.de (J.H.), n.strewinsky@uke.de (N.S.), c.bokemeyer@uke.de (C.B.), g.von-amsberg@uke.de (G.v.A.)

2 Department of Radiotherapy & Radiation Oncology, Hubertus Wald Tumorzentrum – University Cancer Center Hamburg (UCCH), University Medical Center Hamburg-Eppendorf, Martinistrasse 52, 20251 Hamburg, Germany; m.kriegs@uke.de (M.Kr.); k.hoffer@uke.de (K.H.)

3 UCCH Kinomics Core Facility, Hubertus Wald Tumorzentrum – University Cancer Center Hamburg (UCCH), University Medical Center Hamburg-Eppendorf, Martinistrasse 52, 20251 Hamburg, Germany

4 Center of Molecular and Cellular Biology, Bolshoy blvd 30 bld1, 121205 Moscow, Russian Federation; alunit@inbox.ru (O.Y.B.)

5 G.B. Elyakov Pacific Institute of Bioorganic Chemistry, Far Eastern Branch of the Russian Academy of Sciences, Prospekt 100-let Vladivostoku 159, 690022 Vladivostok, Russian Federation; malyarenko-tv@mail.ru (T.V.M.); kicha@piboc.dvo.ru (A.A.K.); ivanchina@piboc.dvo.ru (N.V.I.); stonik@piboc.dvo.ru (V.A.S.)

6 School of Advanced Engineering Studies, Institute of Biotechnology, Bioengineering and Food Systems, Far Eastern Federal University, Russky Island, Ajax Bay, 10, 690922 Vladivostok, Russian Federation

7 Martini-Klinik, Prostate Cancer Center, University Hospital Hamburg-Eppendorf, Martinistrasse 52, 20251 Hamburg, Germany; graefen@uke.de (M.G.)

* Correspondence to: Dr. Sergey A. Dyshlovoy, Laboratory of Experimental Oncology, Department of Oncology, Hematology and Bone Marrow Transplantation with Section Pneumology, University Medical Center Hamburg-Eppendorf, Martinistrasse 52, 20251 Hamburg, Germany; e-mail: s.dyshlovoy@uke.de

Supplementary Table S1. R^2 values (goodness-of-fit) for the dose-response curves used to calculate IC_{50} values. The respective IC_{50} values are presented in Table 1. IC_{50} values were determined using GraphPad Prism software based on MTT assay viability data.

	R² values (goodness-of-fit)									
	**Cancer cells**						**Non-cancer cells**			
	**PC3**	**PC3-DR**	**DU145**	**22Rv1**	**VCaP**	**LNCaP**	**PNT2**	**RWPE-1**	**HEK293T**	**MRC-9**
**PaC**	0.9645	0.9477	0.9701	0.8955	0.9511	0.9872	0.9523	0.9546	0.9426	0.9645
**CuC_1**	0.9807	0.9878	0.9698	0.9551	0.9041	0.9174	0.9828	0.9101	0.9817	0.8254
**CuC_2**	0.9669	0.9310	0.9645	0.9701	0.8955	0.9511	0.9872	0.9523	0.9546	0.9426
**Doce**	0.8792	0.9021	0.8716	0.9345	0.6183	0.9773	N/A	0.7355	0.8779	0.5874
**Cis**	0.8562	0.9118	0.9139	0.935	0.652	0.9658	0.7919	0.9132	0.9788	0.9285

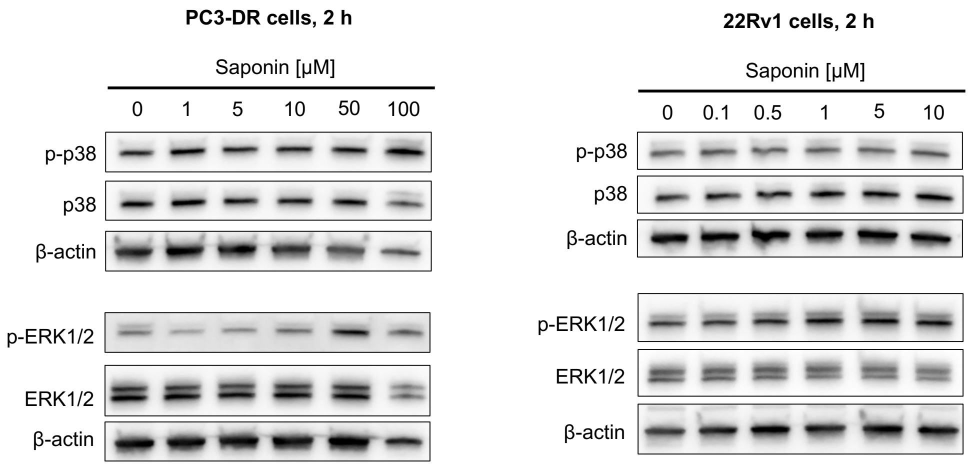

Supplementary Figure S1. Western blot analysis of MAP kinases. 22Rv1 cells were treated with indicated concentrations of saponin (Cat. # 47036-50G-F, Sigma-Aldrich) for 2 h and the MAPK expression was analyzed by Western blotting. β-actin was used as a loading control.

Supplementary Table S2. The effect of combinational treatment with established chemotherapies was evaluated with MTT assay. The cells were exposed to individual drugs of their combinations for 48 h, and the viability was measured by MTT assay. The data are presented as % of effect, where 0 refers to no 0% of dead cells compared to control (i.e. no cytotoxicity effect), 100 refers to 100% dead cells compared to control. Combination of **CuC1 + Cabazitaxel** in **22RV1 cells.**

Drug1	Drug2	Conc1	Conc2	Response	ConcUnit
CuC1	Caba	0	0	0	µM / nM
CuC1	Caba	1	0	95.8597	µM / nM
CuC1	Caba	0.5	0	81.70948	µM / nM
CuC1	Caba	0.25	0	31.10115	µM / nM
CuC1	Caba	0.125	0	0.01	µM / nM
CuC1	Caba	0	2	32.86119	µM / nM
CuC1	Caba	0	1	27.6928	µM / nM
CuC1	Caba	0	0.5	20.35928	µM / nM
CuC1	Caba	0	0.25	16.60171	µM / nM
CuC1	Caba	1	2	98.12262	µM / nM
CuC1	Caba	0.5	2	89.60175	µM / nM
CuC1	Caba	0.25	2	54.9875	µM / nM
CuC1	Caba	0.125	2	49.12068	µM / nM
CuC1	Caba	1	1	96.97719	µM / nM
CuC1	Caba	0.5	1	88.70776	µM / nM
CuC1	Caba	0.25	1	46.08949	µM / nM
CuC1	Caba	0.125	1	33.72963	µM / nM
CuC1	Caba	1	0.5	93.03804	µM / nM
CuC1	Caba	0.5	0.5	85.49498	µM / nM
CuC1	Caba	0.25	0.5	34.8168	µM / nM
CuC1	Caba	0.125	0.5	16.67156	µM / nM
CuC1	Caba	1	0.25	97.49403	µM / nM
CuC1	Caba	0.5	0.25	79.33481	µM / nM
CuC1	Caba	0.25	0.25	32.87516	µM / nM
CuC1	Caba	0.125	0.25	3.275645	µM / nM
CuC1	Caba	0	0	0	µM / nM
CuC1	Caba	1	0	95.07517	µM / nM
CuC1	Caba	0.5	0	82.22889	µM / nM

CuC1	Caba	0.25	0	43.15586	µM / nM
CuC1	Caba	0.125	0	12.97347	µM / nM
CuC1	Caba	0	2	33.19682	µM / nM
CuC1	Caba	0	1	30.02976	µM / nM
CuC1	Caba	0	0.5	24.57327	µM / nM
CuC1	Caba	0	0.25	3.713972	µM / nM
CuC1	Caba	1	2	93.73967	µM / nM
CuC1	Caba	0.5	2	84.35298	µM / nM
CuC1	Caba	0.25	2	52.975	µM / nM
CuC1	Caba	0.125	2	20.74483	µM / nM
CuC1	Caba	1	1	96.61418	µM / nM
CuC1	Caba	0.5	1	81.87276	µM / nM
CuC1	Caba	0.25	1	54.55216	µM / nM
CuC1	Caba	0.125	1	21.8465	µM / nM
CuC1	Caba	1	0.5	95.8256	µM / nM
CuC1	Caba	0.5	0.5	77.53555	µM / nM
CuC1	Caba	0.25	0.5	45.80143	µM / nM
CuC1	Caba	0.125	0.5	23.3268	µM / nM
CuC1	Caba	1	0.25	93.25634	µM / nM
CuC1	Caba	0.5	0.25	71.73565	µM / nM
CuC1	Caba	0.25	0.25	31.77228	µM / nM
CuC1	Caba	0.125	0.25	10.46781	µM / nM

Supplementary Table S2 (continuation). Combination of **CuC1 + Carboplatin** in **22RV1 cells.**

Drug1	Drug2	Conc1	Conc2	Response	ConcUnit
CuC1	Carbo	0	0	0	µM
CuC1	Carbo	1	0	89.09492	µM
CuC1	Carbo	0.5	0	73.42163	µM
CuC1	Carbo	0.25	0	29.94849	µM
CuC1	Carbo	0.125	0	0.01	µM
CuC1	Carbo	0	100	32.93598	µM
CuC1	Carbo	0	50	22.26637	µM
CuC1	Carbo	0	25	11.3613	µM
CuC1	Carbo	0	12.5	0.01	µM
CuC1	Carbo	1	100	98.6755	µM
CuC1	Carbo	0.5	100	89.68359	µM
CuC1	Carbo	0.25	100	54.59897	µM
CuC1	Carbo	0.125	100	46.74025	µM
CuC1	Carbo	1	50	96.52686	µM
CuC1	Carbo	0.5	50	87.28477	µM
CuC1	Carbo	0.25	50	41.17734	µM
CuC1	Carbo	0.125	50	18.54908	µM
CuC1	Carbo	1	25	95.23179	µM
CuC1	Carbo	0.5	25	81.5011	µM
CuC1	Carbo	0.25	25	32.3326	µM
CuC1	Carbo	0.125	25	8.035323	µM
CuC1	Carbo	1	12.5	94.23105	µM
CuC1	Carbo	0.5	12.5	77.42458	µM
CuC1	Carbo	0.25	12.5	21.63355	µM
CuC1	Carbo	0.125	12.5	0.01	µM
CuC1	Carbo	0	0	0	µM
CuC1	Carbo	1	0	94.29944	µM
CuC1	Carbo	0.5	0	77.37372	µM
CuC1	Carbo	0.25	0	34.58461	µM
CuC1	Carbo	0.125	0	3.27482	µM
CuC1	Carbo	0	100	38.66242	µM

CuC1	Carbo	0	50	31.0235	µM
CuC1	Carbo	0	25	13.95813	µM
CuC1	Carbo	0	12.5	4.629436	µM
CuC1	Carbo	1	100	97.27401	µM
CuC1	Carbo	0.5	100	89.42561	µM
CuC1	Carbo	0.25	100	60.83902	µM
CuC1	Carbo	0.125	100	43.10333	µM
CuC1	Carbo	1	50	97.34384	µM
CuC1	Carbo	0.5	50	88.68546	µM
CuC1	Carbo	0.25	50	54.05198	µM
CuC1	Carbo	0.125	50	29.43723	µM
CuC1	Carbo	1	25	97.0366	µM
CuC1	Carbo	0.5	25	85.19419	µM
CuC1	Carbo	0.25	25	44.17864	µM
CuC1	Carbo	0.125	25	20.34019	µM
CuC1	Carbo	1	12.5	94.6765	µM
CuC1	Carbo	0.5	12.5	75.22309	µM
CuC1	Carbo	0.25	12.5	26.75018	µM
CuC1	Carbo	0.125	12.5	6.33318	µM

Supplementary Table S2 (continuation). Combination of **CuC1 + Cisplatin** in **22RV1 cells.**

Drug1	Drug2	Conc1	Conc2	Response	ConcUnit
CuC1	Cis	0	0	0	µM
CuC1	Cis	1	0	94.37126	µM
CuC1	Cis	0.5	0	76.16774	µM
CuC1	Cis	0.25	0	29.33712	µM
CuC1	Cis	0.125	0	1.495558	µM
CuC1	Cis	0	10	40.95414	µM
CuC1	Cis	0	5	26.88984	µM
CuC1	Cis	0	2.5	17.58415	µM
CuC1	Cis	0	1.25	17.44818	µM
CuC1	Cis	1	10	98.13282	µM
CuC1	Cis	0.5	10	90.45864	µM
CuC1	Cis	0.25	10	62.07324	µM
CuC1	Cis	0.125	10	56.61974	µM
CuC1	Cis	1	5	96.98471	µM
CuC1	Cis	0.5	5	83.91746	µM
CuC1	Cis	0.25	5	56.24207	µM
CuC1	Cis	0.125	5	27.95301	µM
CuC1	Cis	1	2.5	95.45894	µM
CuC1	Cis	0.5	2.5	79.58185	µM
CuC1	Cis	0.25	2.5	36.69406	µM
CuC1	Cis	0.125	2.5	10.86168	µM
CuC1	Cis	1	1.25	90.20182	µM
CuC1	Cis	0.5	1.25	69.30932	µM
CuC1	Cis	0.25	1.25	20.84718	µM
CuC1	Cis	0.125	1.25	0.01	µM
CuC1	Cis	0	0	0	µM
CuC1	Cis	1	0	95.79969	µM
CuC1	Cis	0.5	0	72.76779	µM
CuC1	Cis	0.25	0	26.81503	µM
CuC1	Cis	0.125	0	0.309313	µM
CuC1	Cis	0	10	37.60132	µM

CuC1	Cis	0	5	27.90952	µM
CuC1	Cis	0	2.5	11.04802	µM
CuC1	Cis	0	1.25	0.01	µM
CuC1	Cis	1	10	98.49626	µM
CuC1	Cis	0.5	10	92.8176	µM
CuC1	Cis	0.25	10	61.22012	µM
CuC1	Cis	0.125	10	43.59723	µM
CuC1	Cis	1	5	96.38659	µM
CuC1	Cis	0.5	5	85.99686	µM
CuC1	Cis	0.25	5	53.81248	µM
CuC1	Cis	0.125	5	35.39504	µM
CuC1	Cis	1	2.5	94.30865	µM
CuC1	Cis	0.5	2.5	82.31683	µM
CuC1	Cis	0.25	2.5	36.15786	µM
CuC1	Cis	0.125	2.5	10.39767	µM
CuC1	Cis	1	1.25	89.66102	µM
CuC1	Cis	0.5	1.25	73.78297	µM
CuC1	Cis	0.25	1.25	19.26463	µM
CuC1	Cis	0.125	1.25	0.01	µM

Supplementary Table S2 (continuation). Combination of **CuC1 + Docetaxel** in **22RV1 cells.**

Drug1	Drug2	Conc1	Conc2	Response	ConcUnit
CuC1	Doce	0	0	0	µM / nM
CuC1	Doce	1	0	97.07109	µM / nM
CuC1	Doce	0.5	0	72.15752	µM / nM
CuC1	Doce	0.25	0	33.12243	µM / nM
CuC1	Doce	0.125	0	17.34256	µM / nM
CuC1	Doce	0	2	33.10967	µM / nM
CuC1	Doce	0	1	20.92715	µM / nM
CuC1	Doce	0	0.5	14.54887	µM / nM
CuC1	Doce	0	0.25	2.698015	µM / nM
CuC1	Doce	1	2	96.6884	µM / nM
CuC1	Doce	0.5	2	87.79707	µM / nM
CuC1	Doce	0.25	2	48.55787	µM / nM
CuC1	Doce	0.125	2	57.20682	µM / nM
CuC1	Doce	1	1	97.12212	µM / nM
CuC1	Doce	0.5	1	85.99839	µM / nM
CuC1	Doce	0.25	1	43.78692	µM / nM
CuC1	Doce	0.125	1	34.02786	µM / nM
CuC1	Doce	1	0.5	95.64236	µM / nM
CuC1	Doce	0.5	0.5	76.53302	µM / nM
CuC1	Doce	0.25	0.5	46.78471	µM / nM
CuC1	Doce	0.125	0.5	16.52613	µM / nM
CuC1	Doce	1	0.25	95.32344	µM / nM
CuC1	Doce	0.5	0.25	81.11263	µM / nM
CuC1	Doce	0.25	0.25	25.04752	µM / nM
CuC1	Doce	0.125	0.25	0.01	µM / nM
CuC1	Doce	0	0	0	µM / nM
CuC1	Doce	1	0	93.70777	µM / nM
CuC1	Doce	0.5	0	76.9194	µM / nM
CuC1	Doce	0.25	0	30.56712	µM / nM
CuC1	Doce	0.125	0	0.047769	µM / nM
CuC1	Doce	0	2	25.96738	µM / nM

CuC1	Doce	0	1	17.4913	µM / nM
CuC1	Doce	0	0.5	11.17177	µM / nM
CuC1	Doce	0	0.25	0.01	µM / nM
CuC1	Doce	1	2	96.46489	µM / nM
CuC1	Doce	0.5	2	89.28547	µM / nM
CuC1	Doce	0.25	2	47.87416	µM / nM
CuC1	Doce	0.125	2	38.40169	µM / nM
CuC1	Doce	1	1	97.24289	µM / nM
CuC1	Doce	0.5	1	86.89688	µM / nM
CuC1	Doce	0.25	1	49.64854	µM / nM
CuC1	Doce	0.125	1	17.61344	µM / nM
CuC1	Doce	1	0.5	95.82338	µM / nM
CuC1	Doce	0.5	0.5	82.26984	µM / nM
CuC1	Doce	0.25	0.5	37.33706	µM / nM
CuC1	Doce	0.125	0.5	11.70409	µM / nM
CuC1	Doce	1	0.25	93.73507	µM / nM
CuC1	Doce	0.5	0.25	83.25258	µM / nM
CuC1	Doce	0.25	0.25	26.99106	µM / nM
CuC1	Doce	0.125	0.25	0.01	µM / nM

Supplementary Table S2 (continuation). Combination of **CuC1 + Cabazitaxel** in **PC3-DR cells.**

Drug1	Drug2	Conc1	Conc2	Response	ConcUnit
CuC1	Caba	0	0	0	µM / nM
CuC1	Caba	4	0	90.20663	µM / nM
CuC1	Caba	2	0	61.45239	µM / nM
CuC1	Caba	1	0	33.69486	µM / nM
CuC1	Caba	0.5	0	7.995028	µM / nM
CuC1	Caba	0	100	53.44665	µM / nM
CuC1	Caba	0	50	43.53325	µM / nM
CuC1	Caba	0	25	28.06833	µM / nM
CuC1	Caba	0	12.5	26.4286	µM / nM
CuC1	Caba	4	100	93.76474	µM / nM
CuC1	Caba	2	100	87.47374	µM / nM
CuC1	Caba	1	100	79.69306	µM / nM
CuC1	Caba	0.5	100	66.78956	µM / nM
CuC1	Caba	4	50	93.29318	µM / nM
CuC1	Caba	2	50	88.80268	µM / nM
CuC1	Caba	1	50	70.93711	µM / nM
CuC1	Caba	0.5	50	52.89741	µM / nM
CuC1	Caba	4	25	92.65015	µM / nM
CuC1	Caba	2	25	82.57598	µM / nM
CuC1	Caba	1	25	60.59502	µM / nM
CuC1	Caba	0.5	25	38.84983	µM / nM
CuC1	Caba	4	12.5	91.21404	µM / nM
CuC1	Caba	2	12.5	74.39877	µM / nM
CuC1	Caba	1	12.5	42.5687	µM / nM
CuC1	Caba	0.5	12.5	18.3907	µM / nM
CuC1	Caba	0	0	0	µM / nM
CuC1	Caba	4	0	89.15302	µM / nM
CuC1	Caba	2	0	55.30669	µM / nM
CuC1	Caba	1	0	25.10358	µM / nM
CuC1	Caba	0.5	0	5.089928	µM / nM
CuC1	Caba	0	100	58.00786	µM / nM

CuC1	Caba	0	50	46.11695	µM / nM
CuC1	Caba	0	25	33.72617	µM / nM
CuC1	Caba	0	12.5	23.41174	µM / nM
CuC1	Caba	4	100	96.8528	µM / nM
CuC1	Caba	2	100	92.43096	µM / nM
CuC1	Caba	1	100	80.09786	µM / nM
CuC1	Caba	0.5	100	68.36074	µM / nM
CuC1	Caba	4	50	95.72811	µM / nM
CuC1	Caba	2	50	86.82675	µM / nM
CuC1	Caba	1	50	76.48348	µM / nM
CuC1	Caba	0.5	50	53.07375	µM / nM
CuC1	Caba	4	25	95.1129	µM / nM
CuC1	Caba	2	25	80.79959	µM / nM
CuC1	Caba	1	25	60.39181	µM / nM
CuC1	Caba	0.5	25	40.49352	µM / nM
CuC1	Caba	4	12.5	88.58588	µM / nM
CuC1	Caba	2	12.5	69.79304	µM / nM
CuC1	Caba	1	12.5	43.71377	µM / nM
CuC1	Caba	0.5	12.5	20.41258	µM / nM

Supplementary Table S2 (continuation). Combination of **CuC1 + Carboplatin** in **PC3-DR cells.**

Drug1	Drug2	Conc1	Conc2	Response	ConcUnit
CuC1	Carbo	0	0	0	µM
CuC1	Carbo	4	0	83.68378	µM
CuC1	Carbo	2	0	54.30518	µM
CuC1	Carbo	1	0	26.58287	µM
CuC1	Carbo	0.5	0	2.478566	µM
CuC1	Carbo	0	50	9.103727	µM
CuC1	Carbo	0	25	3.970401	µM
CuC1	Carbo	0	12.5	6.120054	µM
CuC1	Carbo	0	6.25	0.01	µM
CuC1	Carbo	4	50	90.47339	µM
CuC1	Carbo	2	50	73.19394	µM
CuC1	Carbo	1	50	45.42464	µM
CuC1	Carbo	0.5	50	26.62986	µM
CuC1	Carbo	4	25	91.85951	µM
CuC1	Carbo	2	25	65.84048	µM
CuC1	Carbo	1	25	38.88171	µM
CuC1	Carbo	0.5	25	8.458224	µM
CuC1	Carbo	4	12.5	90.41466	µM
CuC1	Carbo	2	12.5	58.2051	µM
CuC1	Carbo	1	12.5	30.2596	µM
CuC1	Carbo	0.5	12.5	10.86574	µM
CuC1	Carbo	4	6.25	86.4795	µM
CuC1	Carbo	2	6.25	52.83684	µM
CuC1	Carbo	1	6.25	24.04558	µM
CuC1	Carbo	0.5	6.25	8.363679	µM
CuC1	Carbo	0	0	0	µM
CuC1	Carbo	4	0	93.87076	µM
CuC1	Carbo	2	0	72.89878	µM
CuC1	Carbo	1	0	38.20041	µM
CuC1	Carbo	0.5	0	14.18259	µM
CuC1	Carbo	0	50	33.27609	µM

CuC1	Carbo	0	25	14.8669	µM
CuC1	Carbo	0	12.5	8.386112	µM
CuC1	Carbo	0	6.25	9.352192	µM
CuC1	Carbo	4	50	95.36013	µM
CuC1	Carbo	2	50	91.30797	µM
CuC1	Carbo	1	50	78.29272	µM
CuC1	Carbo	0.5	50	49.69944	µM
CuC1	Carbo	4	25	93.24013	µM
CuC1	Carbo	2	25	79.72842	µM
CuC1	Carbo	1	25	65.22381	µM
CuC1	Carbo	0.5	25	32.18272	µM
CuC1	Carbo	4	12.5	95.27963	µM
CuC1	Carbo	2	12.5	74.40157	µM
CuC1	Carbo	1	12.5	54.30174	µM
CuC1	Carbo	0.5	12.5	19.24109	µM
CuC1	Carbo	4	6.25	92.15328	µM
CuC1	Carbo	2	6.25	57.87087	µM
CuC1	Carbo	1	6.25	20.47553	µM
CuC1	Carbo	0.5	6.25	0.01	µM

Supplementary Table S2 (continuation). Combination of **CuC1 + Cisplatin** in **PC3-DR cells.**

Drug1	Drug2	Conc1	Conc2	Response	ConcUnit
CuC1	Cis	0	0	0	µM
CuC1	Cis	4	0	94.46551	µM
CuC1	Cis	2	0	63.58142	µM
CuC1	Cis	1	0	34.475	µM
CuC1	Cis	0.5	0	10.52382	µM
CuC1	Cis	0	10	50.18962	µM
CuC1	Cis	0	5	29.8412	µM
CuC1	Cis	0	2.5	18.70112	µM
CuC1	Cis	0	1.25	12.79924	µM
CuC1	Cis	4	10	97.94975	µM
CuC1	Cis	2	10	96.78834	µM
CuC1	Cis	1	10	90.47168	µM
CuC1	Cis	0.5	10	72.62385	µM
CuC1	Cis	4	5	97.74828	µM
CuC1	Cis	2	5	91.96492	µM
CuC1	Cis	1	5	69.35293	µM
CuC1	Cis	0.5	5	42.79088	µM
CuC1	Cis	4	2.5	98.12752	µM
CuC1	Cis	2	2.5	82.95805	µM
CuC1	Cis	1	2.5	60.3342	µM
CuC1	Cis	0.5	2.5	27.7791	µM
CuC1	Cis	4	1.25	93.10263	µM
CuC1	Cis	2	1.25	68.31003	µM
CuC1	Cis	1	1.25	36.96374	µM
CuC1	Cis	0.5	1.25	13.84215	µM
CuC1	Cis	0.00	0.00	0	µM
CuC1	Cis	4.00	0.00	92.06339	µM
CuC1	Cis	2.00	0.00	68.90762	µM
CuC1	Cis	1.00	0.00	30.33296	µM
CuC1	Cis	0.50	0.00	5.761505	µM
CuC1	Cis	0.00	10.00	51.54628	µM

CuC1	Cis	0.00	5.00	29.48794	µM
CuC1	Cis	0.00	2.50	21.63034	µM
CuC1	Cis	0.00	1.25	15.79202	µM
CuC1	Cis	4.00	10.00	96.95792	µM
CuC1	Cis	2.00	10.00	93.05107	µM
CuC1	Cis	1.00	10.00	87.16885	µM
CuC1	Cis	0.50	10.00	68.39183	µM
CuC1	Cis	4.00	5.00	96.60675	µM
CuC1	Cis	2.00	5.00	84.10702	µM
CuC1	Cis	1.00	5.00	71.62924	µM
CuC1	Cis	0.50	5.00	37.26443	µM
CuC1	Cis	4.00	2.50	95.93731	µM
CuC1	Cis	2.00	2.50	76.49086	µM
CuC1	Cis	1.00	2.50	50.67931	µM
CuC1	Cis	0.50	2.50	22.91434	µM
CuC1	Cis	4.00	1.25	90.72452	µM
CuC1	Cis	2.00	1.25	65.01174	µM
CuC1	Cis	1.00	1.25	33.9874	µM
CuC1	Cis	0.50	1.25	11.57789	µM

Supplementary Table S2 (continuation). Combination of **CuC1 + Docetaxel** in **PC3-DR cells.**

Drug1	Drug2	Conc1	Conc2	Response	ConcUnit
CuC1	Doce	0	0	0	µM / nM
CuC1	Doce	4	0	92.89235	µM / nM
CuC1	Doce	2	0	61.135	µM / nM
CuC1	Doce	1	0	41.46936	µM / nM
CuC1	Doce	0.5	0	7.259856	µM / nM
CuC1	Doce	0	800	69.53034	µM / nM
CuC1	Doce	0	400	46.20458	µM / nM
CuC1	Doce	0	200	29.98164	µM / nM
CuC1	Doce	0	100	18.68719	µM / nM
CuC1	Doce	4	800	95.40491	µM / nM
CuC1	Doce	2	800	93.65336	µM / nM
CuC1	Doce	1	800	87.00957	µM / nM
CuC1	Doce	0.5	800	77.49082	µM / nM
CuC1	Doce	4	400	95.23579	µM / nM
CuC1	Doce	2	400	90.19859	µM / nM
CuC1	Doce	1	400	79.70139	µM / nM
CuC1	Doce	0.5	400	57.68912	µM / nM
CuC1	Doce	4	200	94.5231	µM / nM
CuC1	Doce	2	200	75.53392	µM / nM
CuC1	Doce	1	200	54.00802	µM / nM
CuC1	Doce	0.5	200	25.18603	µM / nM
CuC1	Doce	4	100	88.95439	µM / nM
CuC1	Doce	2	100	71.0403	µM / nM
CuC1	Doce	1	100	46.80856	µM / nM
CuC1	Doce	0.5	100	17.28595	µM / nM
CuC1	Doce	0	0	0	µM / nM
CuC1	Doce	4	0	96.86168	µM / nM
CuC1	Doce	2	0	65.14164	µM / nM
CuC1	Doce	1	0	24.44336	µM / nM
CuC1	Doce	0.5	0	6.463641	µM / nM
CuC1	Doce	0	800	74.92131	µM / nM

CuC1	Doce	0	400	54.52571	µM / nM
CuC1	Doce	0	200	33.40999	µM / nM
CuC1	Doce	0	100	23.63033	µM / nM
CuC1	Doce	4	800	101.3798	µM / nM
CuC1	Doce	2	800	100.2532	µM / nM
CuC1	Doce	1	800	92.40159	µM / nM
CuC1	Doce	0.5	800	84.86358	µM / nM
CuC1	Doce	4	400	100.6249	µM / nM
CuC1	Doce	2	400	95.92088	µM / nM
CuC1	Doce	1	400	82.7613	µM / nM
CuC1	Doce	0.5	400	60.13024	µM / nM
CuC1	Doce	4	200	99.32402	µM / nM
CuC1	Doce	2	200	86.78003	µM / nM
CuC1	Doce	1	200	65.15326	µM / nM
CuC1	Doce	0.5	200	44.60666	µM / nM
CuC1	Doce	4	100	93.88829	µM / nM
CuC1	Doce	2	100	72.28475	µM / nM
CuC1	Doce	1	100	39.28708	µM / nM
CuC1	Doce	0.5	100	21.77195	µM / nM

Supplementary Figure S2. Original images used for Figure 3.

Uncropped blots for **Figure 3a**

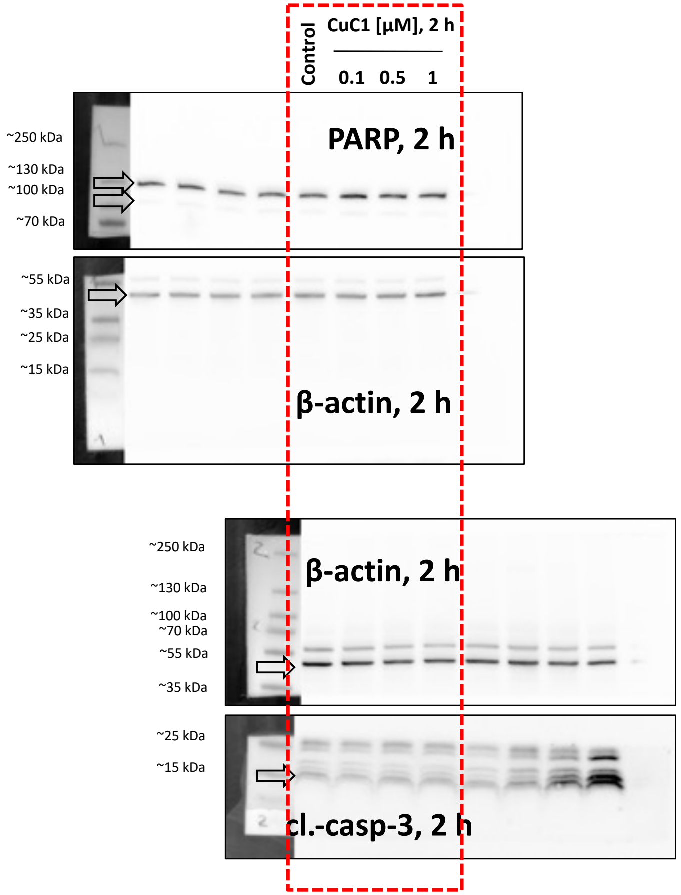

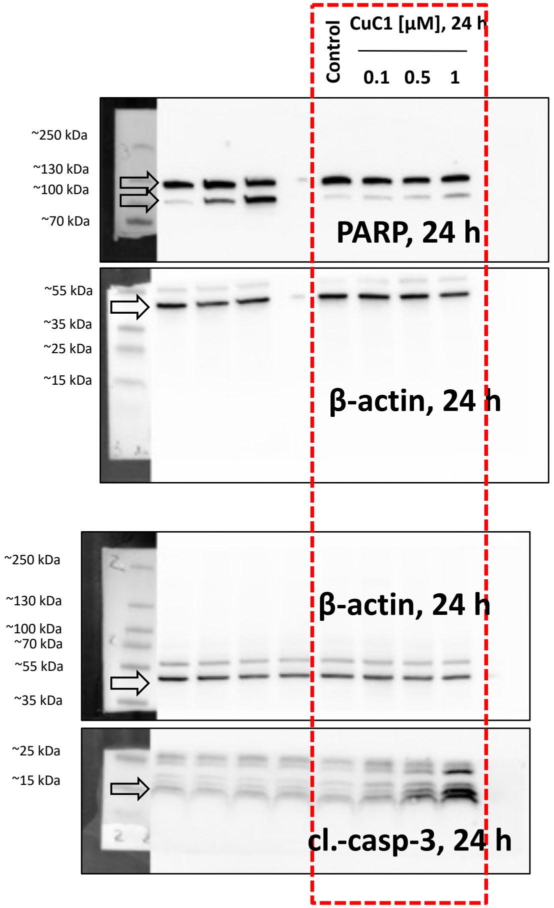

Supplementary Figure S2 (continuation). Original images used for Figure 3.

Uncropped blots for **Figure 3d**

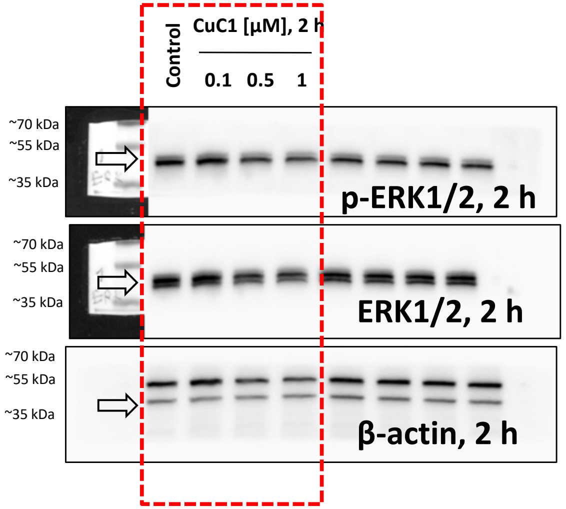

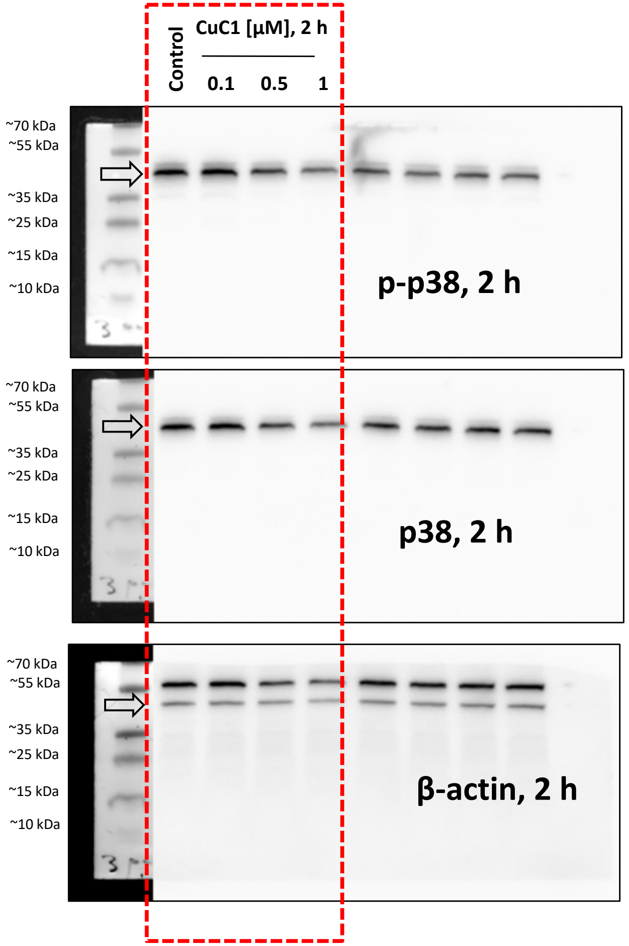

Supplementary Figure S2 (continuation). Original images used for Figure 3.

Uncropped blots for **Figure 3d** (continuation)

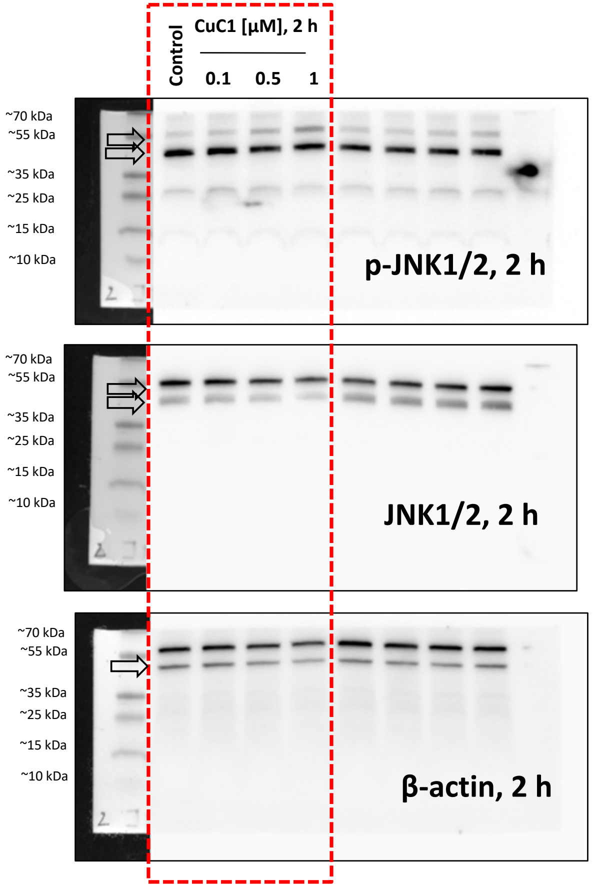

Supplementary Figure S2 (continuation). Original images used for Figure 4.

Uncropped blots for **Figure 4**

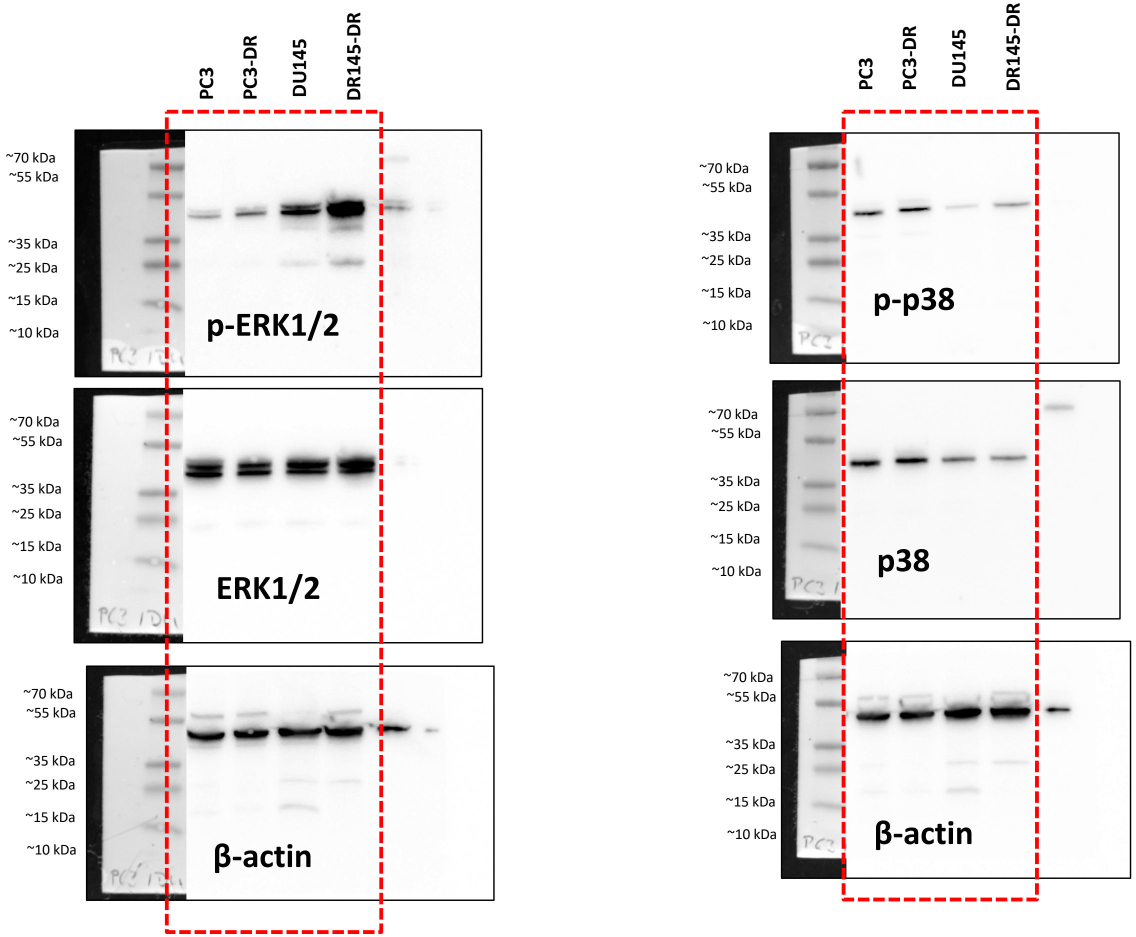

Figure S3. (–)HRESIMS spectrum of pacificusoside C.

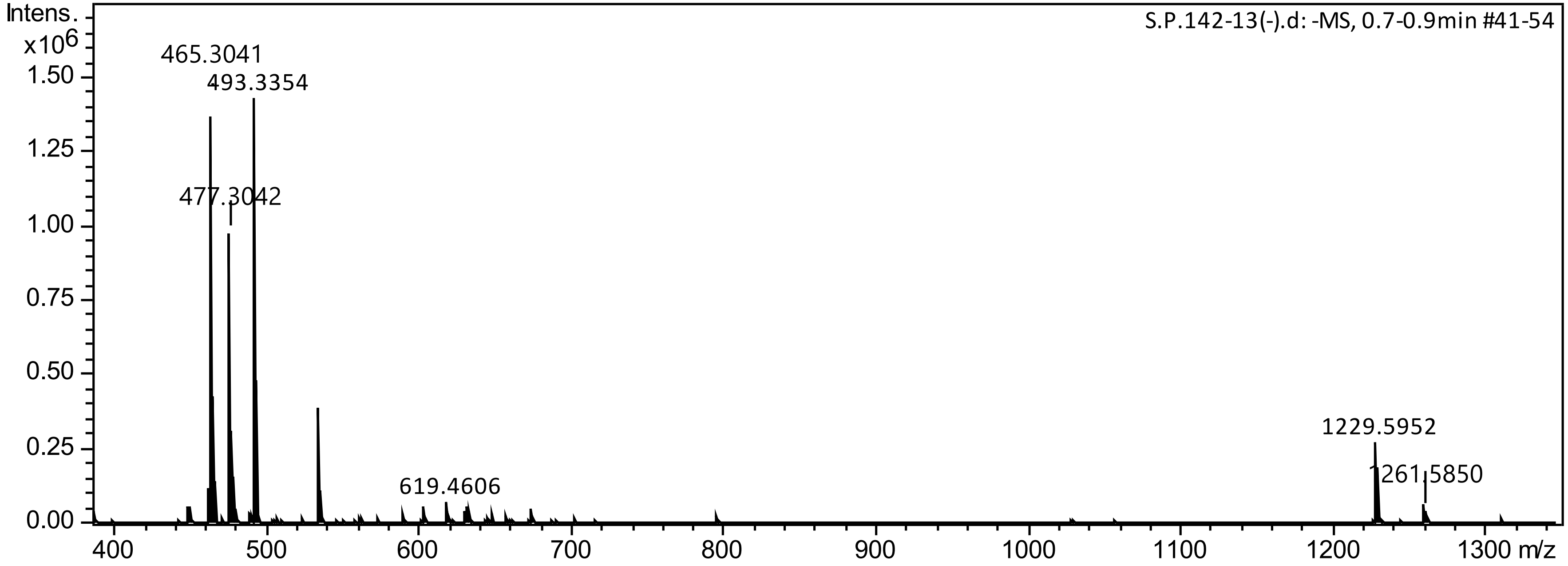

Figure S4. (–)ESIMS/MS spectrum of pacificusoside C.

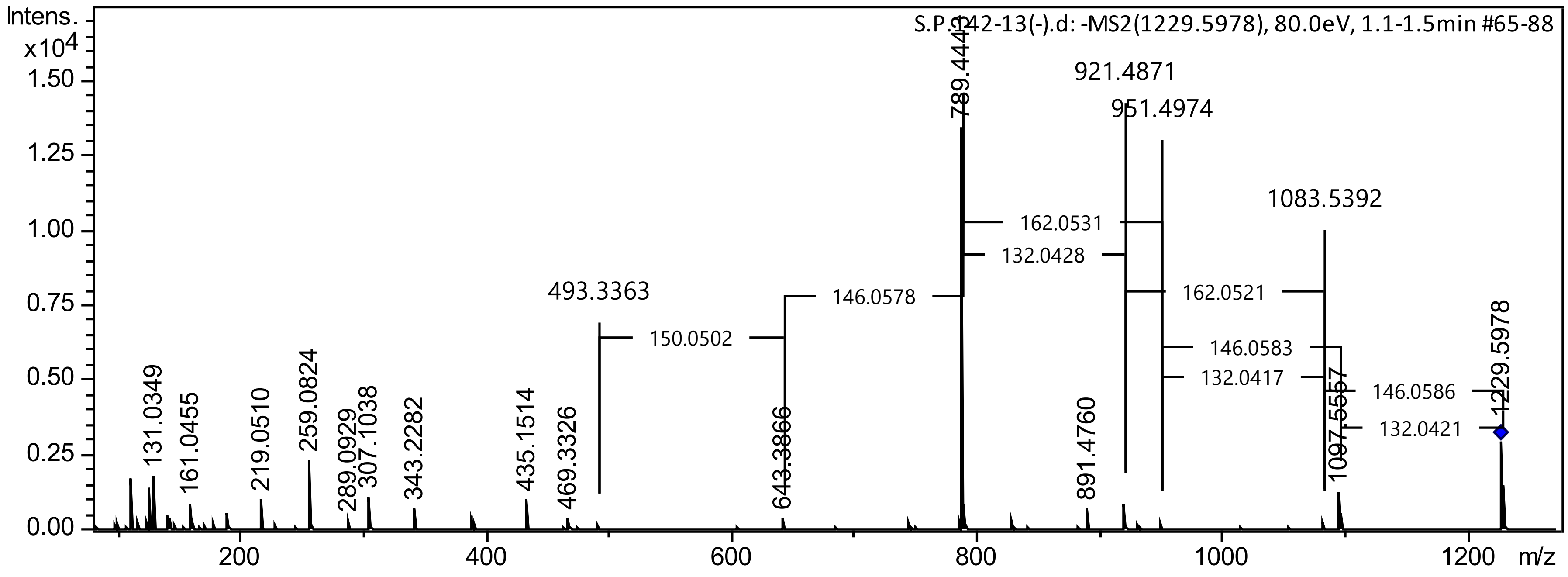

Figure S5. IR spectrum of pacificusoside C in KBr.

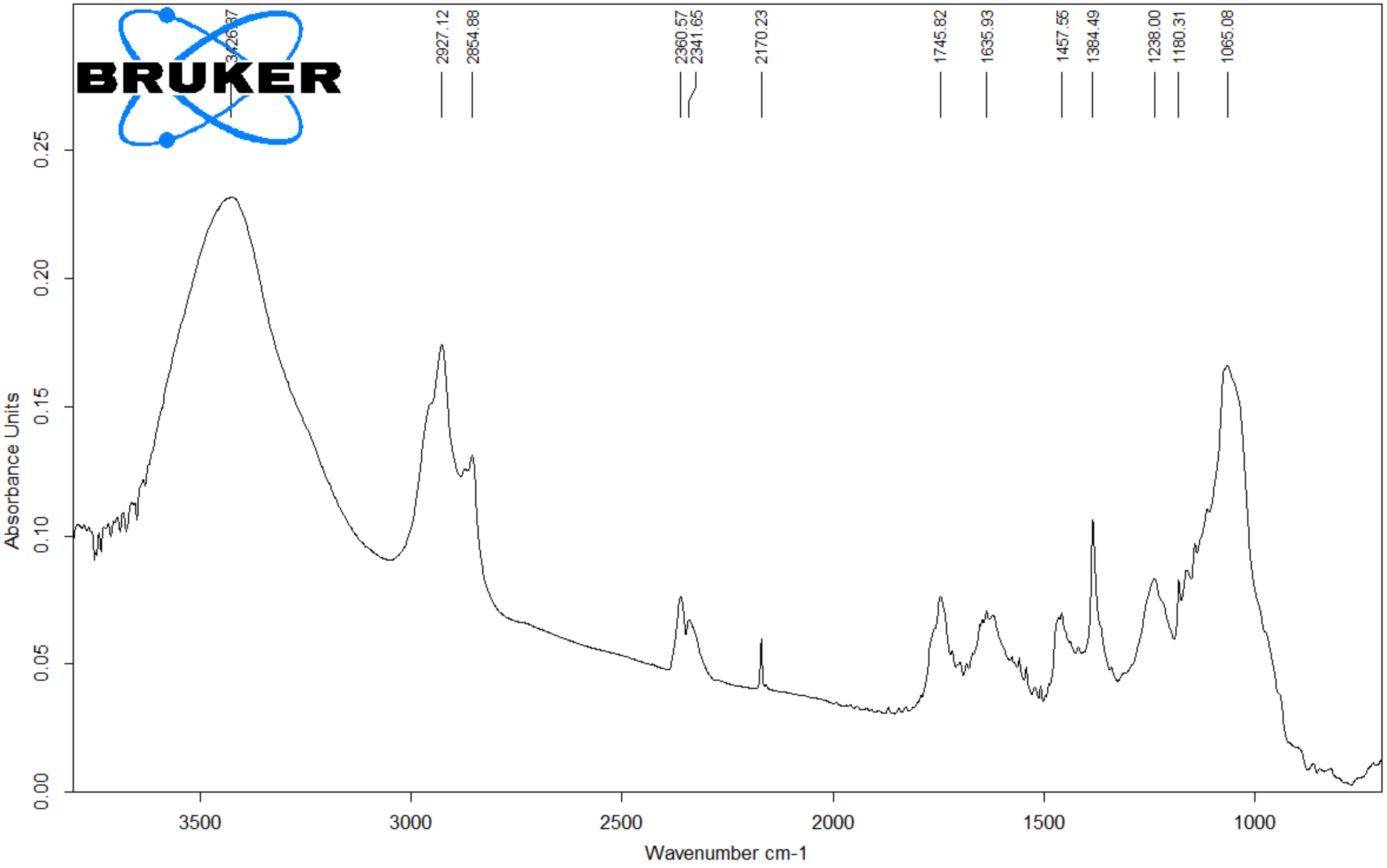

Figure S6. ^1H NMR spectrum of pacificusoside C in C_5D_5N.

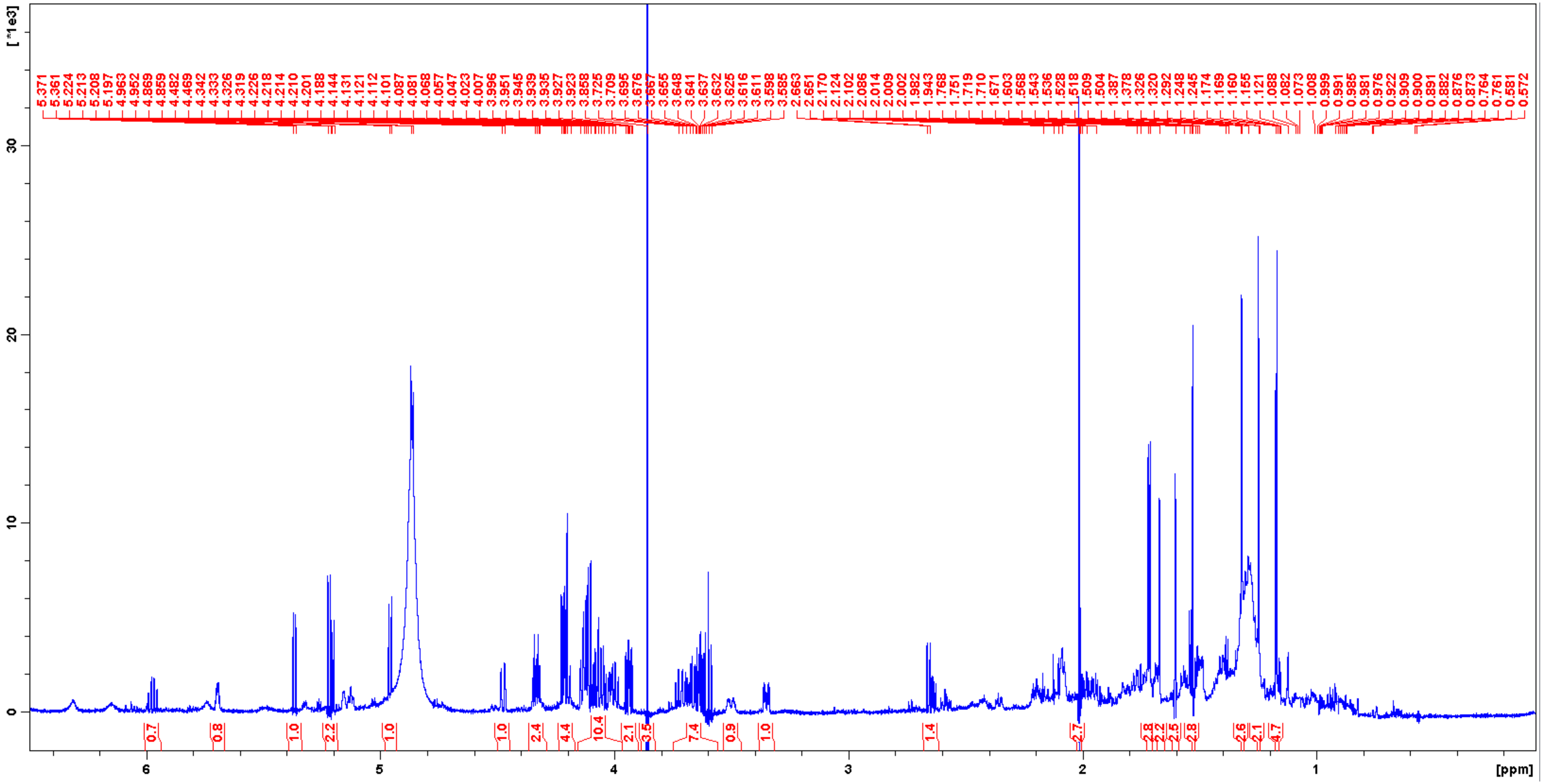

Figure S7. ^{13}C NMR spectrum of pacificusoside C in C_5D_5N.

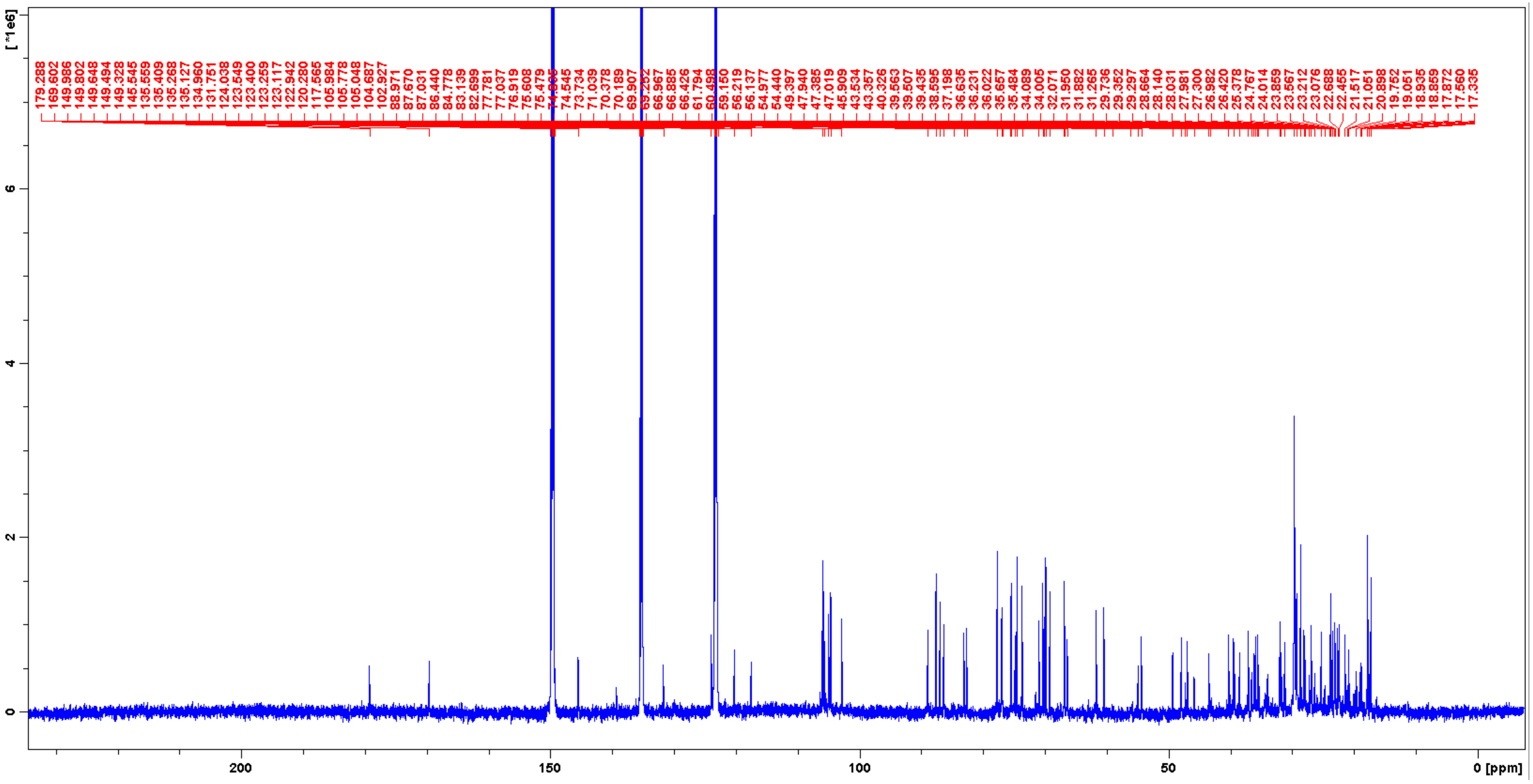

Figure S8. (–)HRESIMS spectrum of cucumarioside C_1 in C_5D_5N.

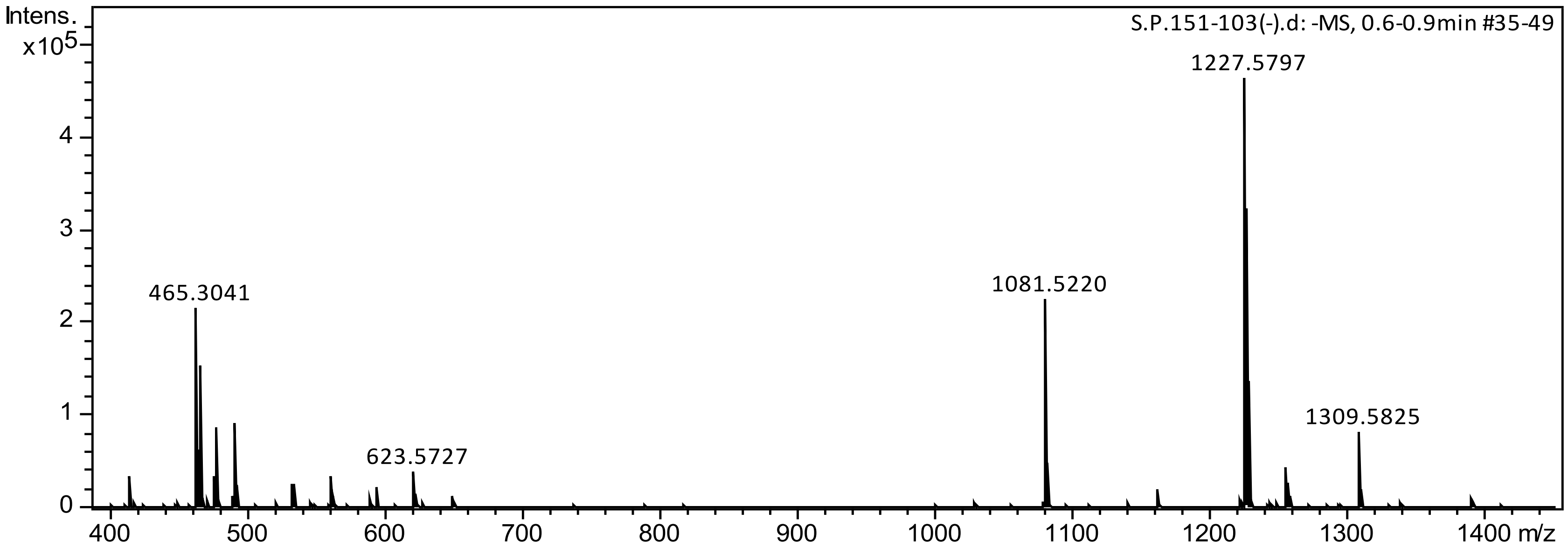

Figure S9. (–)ESIMS/MS spectrum of cucumarioside C_1 in C_5D_5N.

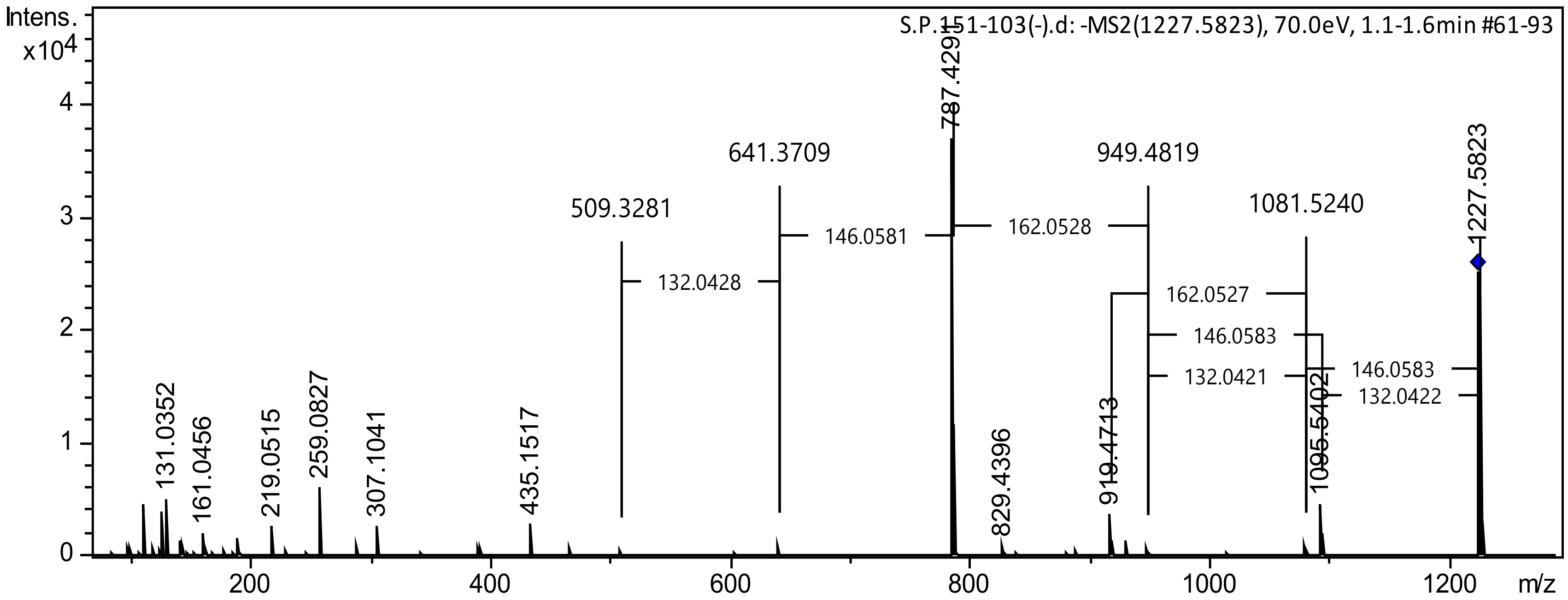

Figure S10. ^{1}H NMR spectrum of cucumarioside C_1 in C_5D_5N.

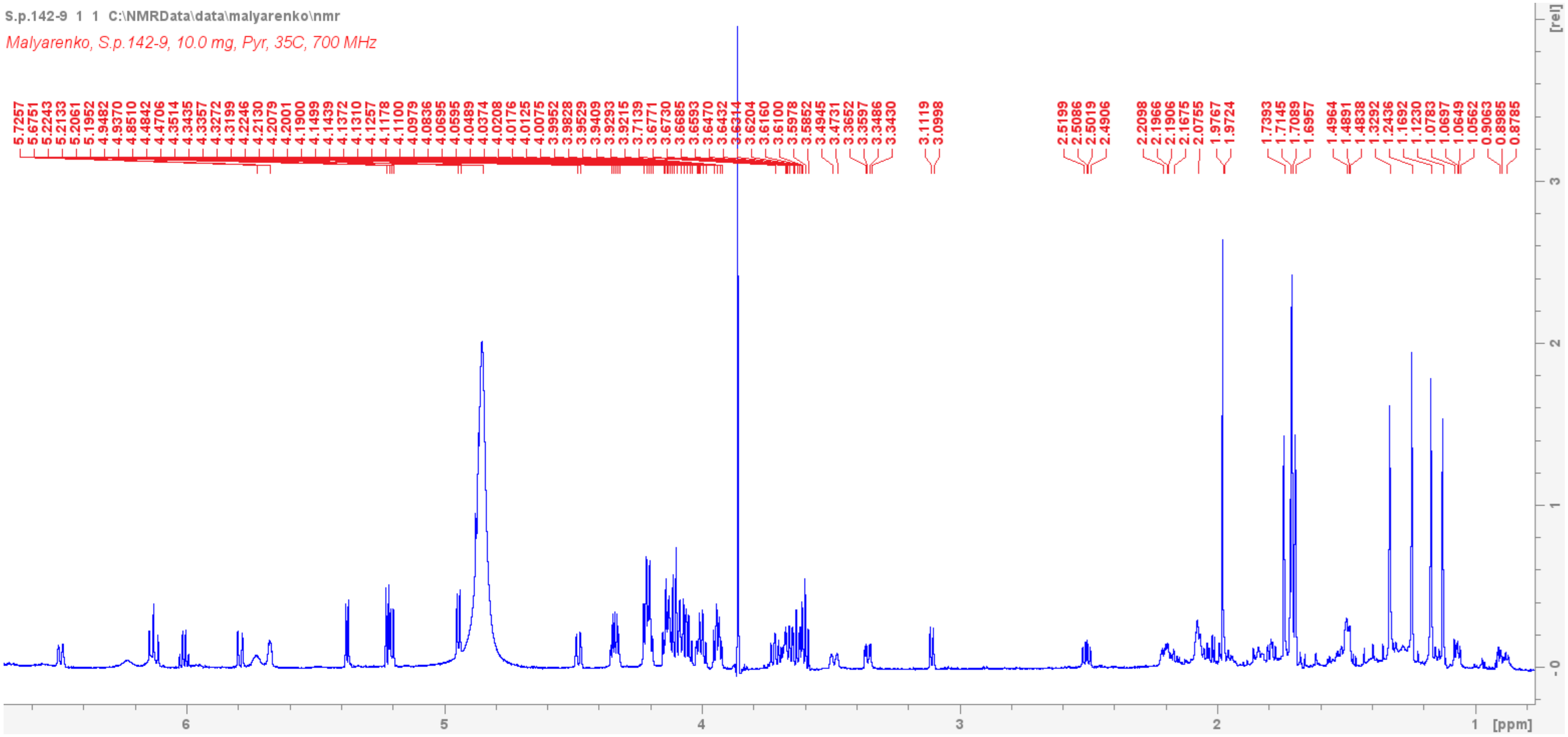

Figure S11. ^{13}C NMR spectrum of cucumarioside C_1 in C_5D_5N.

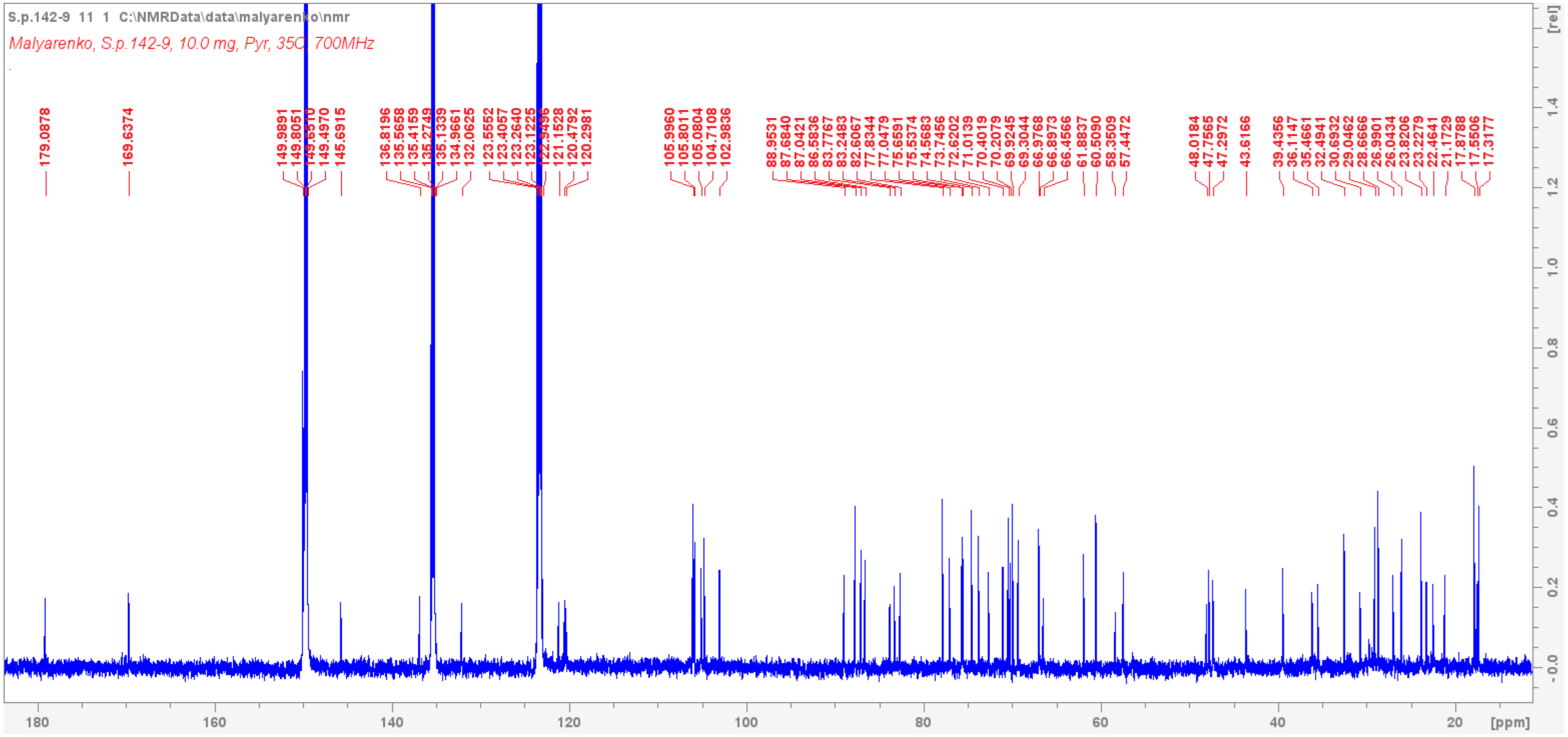

Figure S12. (–)HRESIMS spectrum of cucumarioside C_2 in C_5D_5N.

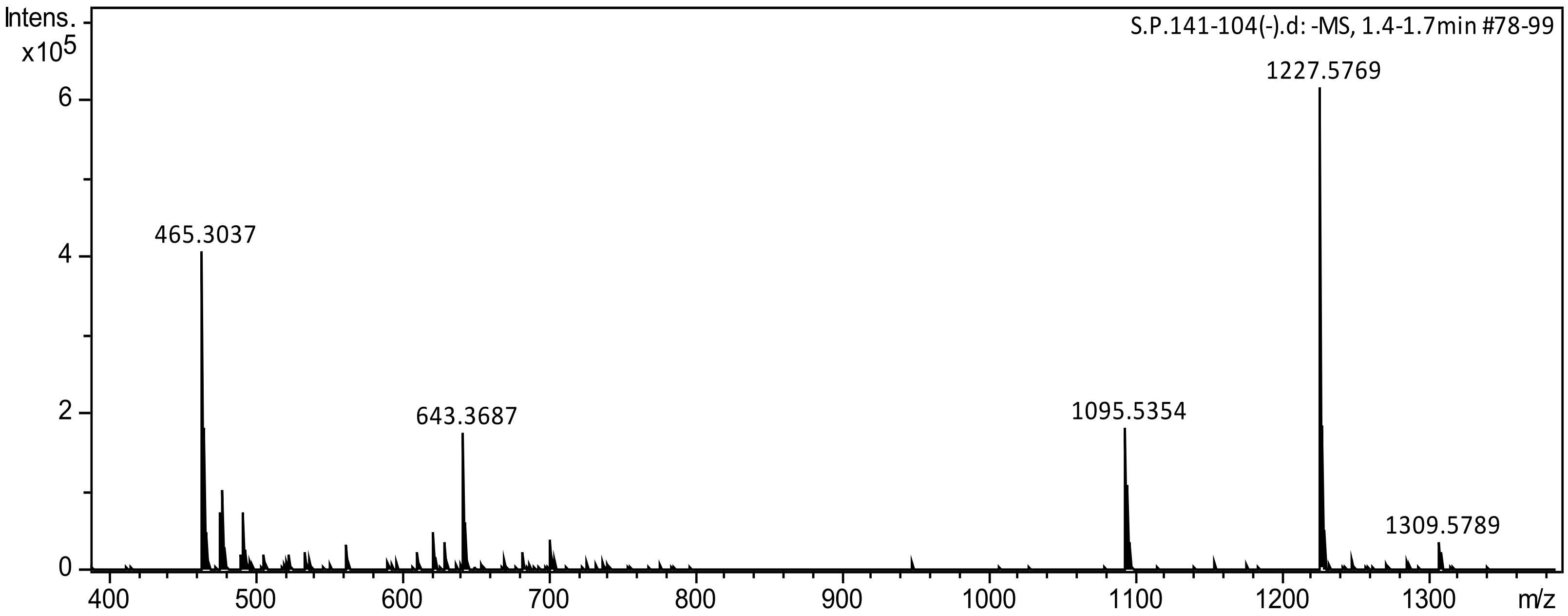

Figure S13. (–)ESIMS/MS spectrum of cucumarioside C_2 in C_5D_5N.

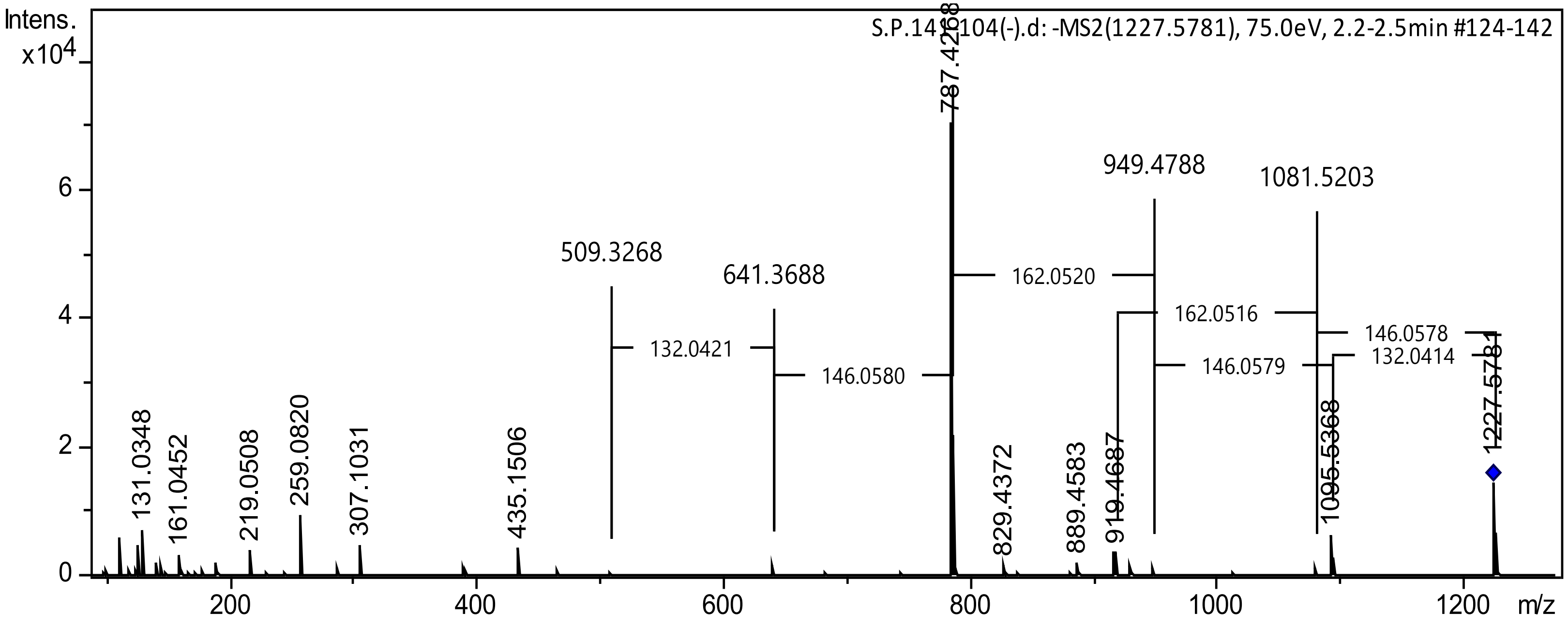

Figure S14. ^{1}H NMR spectrum of cucumarioside C_2 in C_5D_5N.

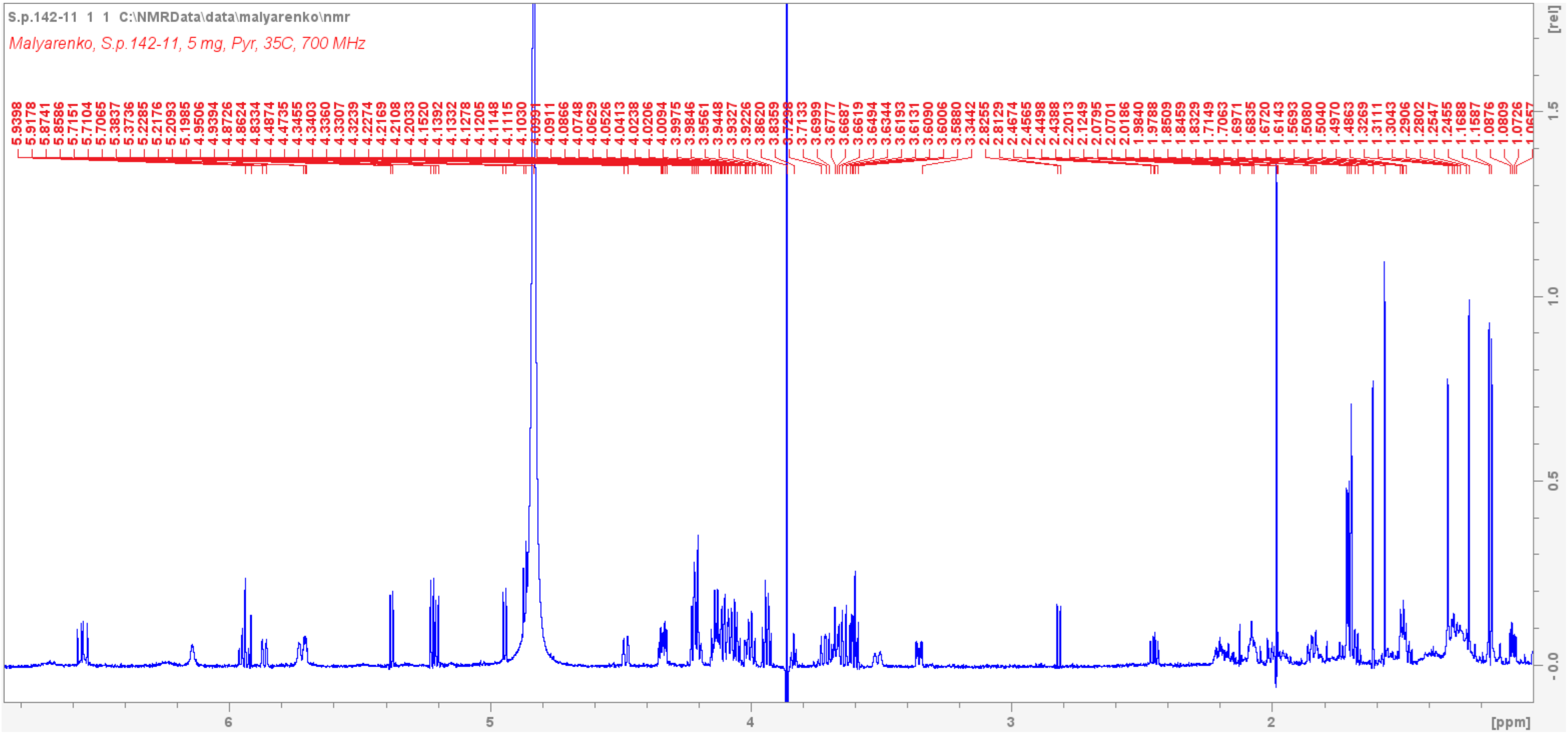

Figure S15. ^{13}C NMR spectrum of cucumarioside C_2 in C_5D_5N.

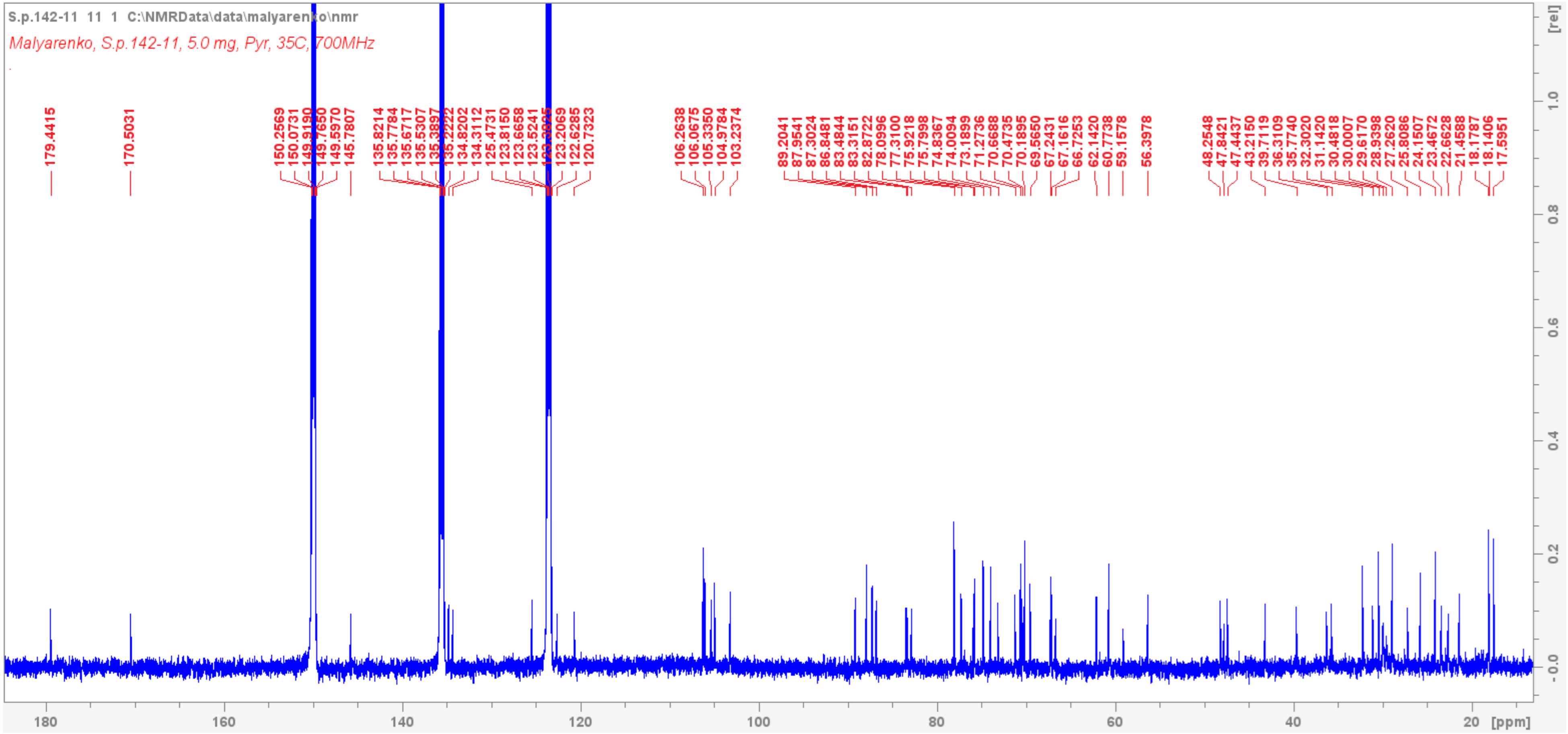

Supplement: Supplementary file 1 — Supplementary Material 1 [file 41598_2025_12914_MOESM1_ESM.pdf]
